# Evaluation of a Model Photo-Caged Dehydropeptide as a Stimuli-Responsive Supramolecular Hydrogel

**DOI:** 10.3390/nano11030704

**Published:** 2021-03-11

**Authors:** Peter J. Jervis, Loic Hilliou, Renato B. Pereira, David M. Pereira, José A. Martins, Paula M. T. Ferreira

**Affiliations:** 1 Center of Chemistry, University of Minho, 4710-057 Braga, Portugal; jmartins@quimica.uminho.pt (J.A.M.); pmf@quimica.uminho.pt (P.M.T.F.); 2 Institute for Polymers and Composites, University of Minho, 4800-058 Guimarães, Portugal; loic@dep.uminho.pt; 3 REQUIMTE/LAQV, Laboratório de Farmacognosia, Departamento de Química, Faculdade de Farmácia, Universidade do Porto, R. Jorge Viterbo Ferreira, n 228, 4050-313 Porto, Portugal; rjpereira@ff.up.pt (R.B.P.); dpereira@ff.up.pt (D.M.P.)

**Keywords:** supramolecular, peptide, dehydropeptide, hydrogel, stimuli-responsive, photo-responsive, carboxy-2-nitrobenzyl, photo-labile, rheology, drug delivery

## Abstract

Short peptides capped on the *N*-terminus with aromatic groups are often able to form supramolecular hydrogels, via self-assembly, in aqueous media. The rheological properties of these readily tunable hydrogels resemble those of the extracellular matrix (ECM) and therefore have potential for various biological applications, such as tissue engineering, biosensors, 3D bioprinting, drug delivery systems and wound dressings. We herein report a new photo-responsive supramolecular hydrogel based on a “caged” dehydropeptide (CNB-Phe-ΔPhe-OH **2**), containing a photo-cleavable carboxy-2-nitrobenzyl (CNB) group. We have characterized this hydrogel using a range of techniques. Irradiation with UV light cleaves the pendant aromatic capping group, to liberate the corresponding uncaged model dehydropeptide (H-Phe-ΔPhe-OH **3**), a process which was investigated by ^1^H NMR and HPLC studies. Crucially, this cleavage of the capping group is accompanied by dissolution of the hydrogel (studied visually and by fluorescence spectroscopy), as the delicate balance of intramolecular interactions within the hydrogel structure is disrupted. Hydrogels which can be disassembled non-invasively with temporal and spatial control have great potential for specialized on-demand drug release systems, wound dressing materials and various topical treatments. Both **2** and **3** were found to be non-cytotoxic to the human keratinocyte cell line, HaCaT. The UV-responsive hydrogel system reported here is complementary to previously reported related UV-responsive systems, which are generally composed of peptides formed from canonical amino acids, which are susceptible to enzymatic proteolysis in vivo. This system is based on a dehydrodipeptide structure which is known to confer proteolytic resistance. We have investigated the ability of the photo-activated system to accelerate the release of the antibiotic, ciprofloxacin, as well as some other small model drug compounds. We have also conducted some initial studies towards skin-related applications. Moreover, this model system could potentially be adapted for on-demand “self-delivery”, through the uncaging of known biologically active dehydrodipeptides.

## 1. Introduction

**Figure 1:**
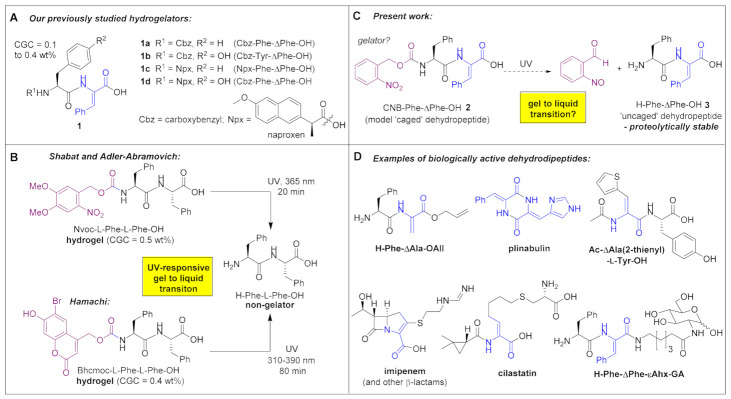
Panel (**A**): Our previously reported aromatic-capped dehydrodipeptide hydrogelators **1a**–**d**. Panel (**B**): Previous UV-responsive peptide hydrogel system based on the photo-cleavage of Ar-Phe-Phe-OH to H-Phe-Phe-OH. Panel (**C**): Our target CNB-capped peptide **2** and how it might act as a photo-responsive hydrogel for the light-triggered release of dehydrodipeptide **3**. Panel (**D**): Structures of known biologically active dehydrodipeptides—the system illustrated in Panel C could potentially be adapted for the release of therapeutic molecules. H-Phe-ΔAla-OAll is a cathepsin B inhibitor, plinabulin is in phase 3 clinical trials for non-small cell lung cancer, Ac-ΔAla(2-thienyl)-Tyr-OH activates macrophages for the selective killing of cancer cells. Imipenem/cilastatin in combination is a wisely used antibiotic. H-Phe-ΔPhe-εAhx-GA is an antimicrobial compound [36,37,38,39,40].

Short peptides capped with aromatic groups attached at the *N*-terminus often have the ability to self-assemble into supramolecular hydrogels, by trapping water molecules within a highly ordered three-dimensional molecular network [1]. The self-assembly process is governed by non-covalent linkages such as hydrogen bonds, van der Waals and π-stacking interactions [2]. In contrast to other types of hydrogelator, such as physically and chemically cross-linked polymers, these hydrogelators are low in molecular weight, easily synthesized on scale and their mechanical properties can be readily tuned [3]. The by-products of metabolic degradation are simple amino acids and therefore inherently non-toxic. Gel formation is initiated by effecting a decrease in solubility to a homogeneous solution of hydrogelator, which can be achieved through a temperature change [4], pH change [5] or enzymatic modification [6]. Peptide hydrogels have found many medicinal applications such as drug delivery vehicles in targeted therapies against cancer and inflammatory diseases [7], anti-bacterial wound dressings [8], 3D bioprinting, tissue engineering [9], skin regeneration, and as extracellular culture medium for the culturing of stem cells and neuronal cells [10]. The peptide capping groups are usually bi- or tricyclic aromatic groups, such as fluorenylmethoxycarbonyl (Fmoc), 9-anthracenemethoxycarbonyl (Amoc), indole-3-acetyl, naphthoyl or naproxen [11]. In our laboratory, we have found that various dehydrodipeptides capped on the *N*-terminus with either naproxen (an NSAID drug) or a carboxybenzyl (Cbz or Z) group (e.g., **1a**–**d**, Figure 1A), are capable of forming stable hydrogels at concentrations as low as 0.1% [12,13,14,15,16,17]. The presence of the dehydroamino acid residue provides proteolytic resistance and greater conformational rigidity [13,14,15,16,17]. These hydrogels are generally non-cytotoxic and some show excellent drug encapsulation and release properties.

In the area of “smart” materials, stimuli-responsive supramolecular peptide hydrogels, where gel disassembly can be triggered when required by means of a remote stimulus, are attracting attention owing to their potential biological applications in controlled drug release [18,19], conversion of self-assembling prodrugs [20,21,22], anti-microbial wound treatment [23,24], dissolution-on-demand wound dressings [25,26], and tunable cell culture platforms [27]. The non-invasive stimulus for gel dissolution can include treatment with UV light [28,29], the application of a magnetic field [30,31], the pH at the desired release site [32,33], or enzymatic degradation [34,35]. In the case of UV-responsive hydrogel systems, a common approach is to switch the aromatic capping group that is required for self-assembly, for a photo-cleavable version of the capping group. Upon a short exposure of the hydrogel to UV light, the capping group is cleaved, releasing a peptide that is more aqueous-soluble than the starting material, accompanied by a loss of π-π stacking interactions. The nanostructures of the hydrogel network are disrupted, ultimately leading to a gel to sol, or gel to solution, transition. This premise was explored by Hamachi [29] and later by Shabat and Adler-Abramovich [18]. Hamachi found that diphenylalanine capped on the *N*-terminal with a photo-cleavable coumarin derivative (Bhcmoc-Phe-Phe-OH) could form a hydrogel with a critical gelation concentration (CGC) of 0.4 wt.%, which underwent a gel to sol transition within 80 min upon exposure to UV light (310–390 nm) (Figure 1B) [29]. Hamachi later adapted this system towards a two-photon responsive system [28]. Adler-Abramovich studied a similar photo-responsive system, featuring a nitroveratryloxycarbonate-capped dipeptide, Nvoc-Phe-Phe-OH (Figure 1B) [18]. This compound was able to form a hydrogel (CGC = 0.5 wt.%) which underwent a gel to sol transition in 20 min upon UV exposure (365 nm). Furthermore, the gel to sol transition could be employed to accelerate the release of a model dye compound from the hydrogel network.

Our research group is interested in photo-responsive peptide hydrogel systems based on dehydrodipeptides, for targeted on-demand drug delivery and other applications. This can be envisaged as either the delivery of existing drugs such as anti-inflammatory, anti-cancer and antimicrobial compounds, or the potential “self-delivery” of one of the many biologically active dehydrodipeptide compound known in the literature [36,37,38,39,40]. We chose to investigate a model system, based on a carboxy-2-nitrobenzyl-capped (CNB-capped) dehydrodipeptde (CNB-Phe-ΔPhe-OH **2**) (Figure 1C). Although based on a similar principle, this hydrogel system has some key differences to the systems described by Hamachi [29] and Adler-Abramovich [18]. Firstly, both the putative hydrogelator, CNB-Phe-ΔPhe-OH (**2**), and its UV-deprotection product, H-Phe-ΔPhe-OH (**3**), are expected to be resistant to action of endogenous peptidases [13,14,15,16,17,41,42]. Both our own group, and the group of Chauhan, have demonstrated the stability of the -Phe-ΔPhe- motif compared to the canonical dipeptide—-Phe-Phe- motif [41]. In the wider scope, this increased enzymatic stability would also be present in modified systems involving the uncaging of biologically active dehydrodipeptides (Figure 1D) [13,14,15,16,17,41,42]. Secondly, the UV-deprotection compound in this case is H-Phe-ΔPhe-OH (**3**), which, rather unusually for a dipeptide lacking a capping group, is already known to be a hydrogelator under certain conditions, as are some other uncapped dehydropeptides [41,42]. Therefore, it could not be presumed that the photolysis of CNB-L-Phe-*Z*-ΔPhe-OH (**2**) would definitely effect a gel to sol transition. Thirdly, here we have chosen the CNB group as the photo-labile capping group, to be complementary to the capping groups used in the previously UV-responsive systems. CNB is a commonly employed photo-caging group for studying biological systems and can be photolytically removed with 360 nm UV light [43,44]. CNB is known to cleave at a slower rate than Bhcmoc and Nvoc groups, which although could be a disadvantage in a “real” system, in this model system it may increase the window for studying a partially-degraded hydrogel network.

To this end, CNB-Phe-ΔPhe-OH (**2**) was synthesized in order to investigate (1) its ability to form a hydrogel and (2) if the putative hydrogel is photo-responsive (i.e., can undergo a gel to liquid transition on-demand). Hydrogel properties were characterized by rheometry, fluorescence spectroscopy and STEM microscopy, and compared with those of the related (but not UV labile) hydrogels **1a** and **1b** (Figure 1A). The gel-to-sol transition upon UV irradiation was assessed visually by tube inversion tests and also using STEM microscopy to show that hydrogel fibrils formed within the fibers of a model cotton gauze dressing are dissolved upon UV irradiation. HPLC and ^1^H NMR were used to follow the time-course of the conversion of **2** in its gel state into dipeptide **3**. This gel-to-sol transition was also assessed indirectly using fluorescence spectroscopy. The effect of a short exposure to UV light (360 nm) on the drug delivery properties of hydrogel **2** was studied. The cytotoxicity was assessed, and an initial in vitro experiment was performed, examining the potential skin-healing properties of photo-generated dipeptide **3** in a scratch assay.

## 2. Materials and Methods

### 2.1. Synthetic Chemistry

^1^H and ^13^C NMR spectra were recorded on a Bruker Avance III (Bruker BioSpin Corp., Billerica, MA, USA) at 400 and 100.6 MHz, respectively. DEPT θ 45° and 135°, HMQC and HMBC were used to attribute some signals. Chemical shifts (δ) are reported in parts per million (ppm) and coupling constants (J) are reported in Hertz (Hz). Petroleum ether refers to the boiling range of 40–60 °C. Acetonitrile was dried over silica and calcium hydride (CaH_2_) and stored over molecular sieves. High resolution mass spectrometry (HRMS) data were recorded by the mass spectrometry service of the University of Vigo, Spain. The synthetic procedures and characterization data for compounds **4**–**10** are reported in the Supporting Information. All canonical amino acids are present as the L stereoisomer. Dehydroamino acids are present as the *Z* geometric isomer.

#### 2.1.1. CNB-Phe-ΔPhe-OH (2)

The methyl ester **7** (500 mg, 0.98 mmol) was dissolved in dioxane (9.8 mL) and a solution of NaOH 1 M (1.47 mL, 1.47 mmol, 1.5 equiv) was added. The reaction was followed by TLC until no starting material was detected (typically 5 h). The organic solvent was removed under reduced pressure, and the reaction mixture was acidified to pH 3 with KHSO_4_ solution (1 M). The solid was filtered and washed with Et_2_O. The solid was identified as CNB-Phe-ΔPhe-OH (**2**) (279 mg, 58%). ^1^H NMR (DMSO-d6, 400 MHz) δ 2.81 (1H, dd, J = 13.6, 11.2, β-*H*_A_H_B_Ph), 3.15 (1H, dd, J = 13.6, 3.6, β-H_A_*H*_B_Ph), 4.40–4.47 (1H, m, α-C*H*CH_2_Ph), 5.26–5.35 (2H, m, C*H*_2_ of CNB, 7.17–7.42 (9H, m, Ar*H*, β-CH ΔPhe, N*H* Phe), 7.46 (1H, d, J = 7.6, Ar*H*), 7.47-7.58 (3H, m, Ar*H*), 7.68 (1H, dd, J = 9.2, 8.8, Ar*H*), 7.87 (1H, d, J = 9.0, Ar*H*), 8.09 (1H, dd, J = 9.0, 0.8, Ar*H*), 9.67(1H, s, N*H* ΔPhe); ^13^C NMR (DMSO-d6, 100 MHz) δ 37.0 (CH_2_, β-C*H*_2_ of Phe), 56.6 (CH, α-C*H* of Phe), 62.1 (CH_2_, C*H*_2_ of CNB), 124.8 (CH, Ar), 126.4 (CH, Ar), 127.4 (C, α-C ΔPhe), 128.2 (CH, Ar), 128.3 (CH, Ar), 128.4 (CH, Ar), 128.8 (CH, Ar), 128.9 (CH, Ar), 129.4 (CH, Ar), 129.9 (CH, Ar), 133.1 (C, Ar), 134.1 (C, Ar), 134.2 (CH, β-CH ΔPhe), 138.2 (C, Ar), 146.9 (C, Ar), 155.7 (C, C=O), 166.8 (C, C=O), 171.0 (C, C=O); HRMS *m/z* (EI) 490.1609 ([M+H]^+^). C_26_H_24_N_3_O_7_ requires 490.1614.

#### 2.1.2. H-Phe-ΔPhe-OH (3)

*Method 1 (from Boc-Phe-ΔPhe-OMe (**10**)).* Compound **14** (423 mg, 0.99 mmol) was dissolved in dioxane (9.90 mL) and a solution of NaOH 1 M (1.49 mL, 1.49 mmol, 1.5 equiv) was added. The reaction was followed by TLC until no starting material was detected (typically 5 h). The organic solvent was removed under reduced pressure, and the reaction mixture was acidified to pH 3 with KHSO_4_ (1 M). The solid was filtered and washed with Et_2_O. After removal of the residual Et_2_O, the solid was dissolved in TFA (3.0 mL) and the reaction mixture was stirred at room temperature for 30 min. The TFA was then removed under reduced pressure. Traces of residue TFA were removed by the addition and removal of CHCl_3_ (3 × 10 mL) under reduced pressure, affording H-Phe-ΔPhe-OH (**3**) as a white solid (237 mg, 54%).

*Method 2 (from CNB-Phe-ΔPhe-OH (**2**)).* A solution of compound **2** (100 mg, 0.204 mmol) in MeCN (5 mL) was irradiated with UV light (360 nm) for 3 h. After cooling to 0 °C, the precipitate formed was filtered and the solid was then washed with cooled (0 °C) MeCN (2 × 5 mL). Removal of the residual MeCN under reduced pressure afforded H-Phe-ΔPhe-OH (**7**) as a white solid (48 mg, 76%). The ^1^H NMR and ^13^C NMR data were identical to that from the material obtained from method 1.

^1^H NMR (DMSO-d_6_, 400 MHz) δ 2.92 (1H, dd, J = 13.9, 7.0, β-C*H*_A_CH_B_Ph Phe), 3.06 (1H, dd, J = 13.9, 5.2, β-CH_A_C*H*_B_Ph Phe), 4.35-4.41 (1H, m, α-C*H* Phe), 6.58 (1H, d, J = 7.6, N*H*_2_), 6.94 (1H, s, β-C*H* ΔPhe), 7.12-7.7.41 (8H, m, Ph*H*), 7.52 (2H, d, J = 6.8, Ph*H*), 7.98 (1H, d, s, N*H* ΔPhe); ^13^C NMR (DMSO-d_6_, 100 MHz) δ 37.4 (CH_2_, β-C*H*_2_ Phe), 53.8 (CH, α-CI Phe), 126.5 (CH, Ph), 126.7 (CH, β-C*H* ΔPhe), 128.2 (CH, Ph), 128.3 (CH, Ph), 128.4 (CH, Ph), 129.4 (CH, Ph), 129.5 (CH, Ph), 134.3 (C, Ph), 137.2 (C, Ph), 154.7 (C, α-C ΔPhe), 167.1 (C, C=O), 173.4 (C, C=O); HRMS *m/z* (EI) 311.1388. C_18_H_19_N_2_O_3_ requires 311.1396.

### 2.2. Gel Preparation and Determination of Critical Gelation Concentration

NaOH (1.0 M) was added to a mixture of hydrogelator **2** (3.0, 4.0, 5.0, and 6.0 mg) in water (1.0 mL) in a small glass vial, until pH 10 was reached (~30 μL) and the resulting mixture was sonicated for ~1 min until dissolved, to afford concentrations of 0.3, 0.4, 0.5, and 0.6 wt.% respectively. GdL (3.0 mg) was added to each vial, followed by thorough mixing for 1 min. The solutions were left to stand overnight. The CGC was then determined through tube inversion tests, and was found to be 0.4 wt.%.

### 2.3. Gel-to-Sol Transition of the Gel of 6 under UV Irradiation

A hydrogel of **2** was prepared in a UV cuvette, as described in Section 2.2, at 0.4 wt.%. The sample was irradiated with UV light (360 nm) for 3 h, and a tube inversion test was made and photographed (after 5 min inversion) every 30 min. As a control, this same procedure was also performed on a hydrogel made from **1a** at 0.4 wt.%.

In the presence of simulated body fluid (SBF): Three separate hydrogels of **2** were prepared in UV cuvettes as described in Section 2.2, at 0.4 wt.%. After allowing to stand overnight, 1.0 mL of SBF solution was carefully added to the surface of each hydrogel and left to stand for 1 h. One hydrogel was irradiated with UV light (360 nm) for 20 min, one for 10 min, and one with no UV irradiation. After 3 h, a tube inversion test was performed on each sample.

### 2.4. Studying Photo-Cleavage of 6 to Dipeptide 7 in the Gel State by HPLC 

A hydrogel of **2** was prepared in a UV cuvette, as described in Section 2.2, at 0.4 wt.%. The sample was irradiated with UV light (360 nm) for 3 h, during which time aliquots (10 μL) were taken at 10, 30, 70, 90, 110, 140, and 180 min. Each aliquot was further diluted with MeCN (250 μL), and then analyzed by HPLC (40:60:1 H_2_O/MeCN/TFA). The percentage consumption of compound **2**, and the percentage formation of compound **3**, were plotted against time.

### 2.5. Studying Photo-Cleavage of 2 in Diluted Version of the Gel State by ^1^H NMR

NaOD (1.0 M) was added to a mixture of hydrogelator **2** (4.0 mg) in D_2_O (1.0 mL) in a small glass vial, until pH 10 was reached (~30 μL) and the resulting mixture was sonicated for ~1 min until dissolved, to afford concentrations of 0.3 wt.%. GdL (4.0 mg) was added to the vial, followed by thorough mixing for 1 min. The solution was then transferred to an NMR tube and left to stabilize overnight. The sample was then irradiated with UV light (360 nm), and ^1^H NMR spectra were recorded at 1, 5, 10, 25, and 40 min intervals. The degree of photo-cleavage of the CNB group at each time point was determined through the percentage integration loss of the *o*-nitrobenzylic CH_2_ proton resonance. The percentage of photo-cleavage was then plotted against time.

### 2.6. Rheology

The viscoelastic characterization of gels was performed with a stress-controlled rotational rheometer Anton Paar MCR300 (Anton Paar GmbH, Graz, Austria). Liquid samples were loaded into the Couette geometry of the rheometer and temperature was kept at 25 °C during testing. After a three-hour rest period ensuring gel setting and structural equilibrium of samples, mechanical spectra of gels were recorded using a fixed strain amplitude (values ranging from 10^−4^ to 10^−2^% depending on the gel elasticity) and ramping the frequency from 100 Hz down to 0.01 Hz. Finally, a sweep in the strain amplitude was performed from 0.001% to 500%, to assess the linear regime of viscoelasticity and the large amplitude oscillatory strain (LAOS) regime.

### 2.7. Assessment of the pH Dependence on Self-Assembly by Fluorescence Spectroscopy

Hydrogelator **2** (1.0 mg) was dissolved in EtOH (5 mL) to afford a 0.40 mM solution. From this solution, 10 μL was taken and added to a solution of buffer (0.990 mL) at the required pH (2, 3, 4, 5, 6, 8, and 10) (previously prepared from a sodium phosphate 0.1 M solution (0.1 M) and a mixed solution of citric acid (0.05 M) and boric acid (0.2 M), as described by Perrin and Dempsey) [45] to afford solutions of 4.0 μM. The emission spectrum was obtained for each hydrogelator solution to evaluate the influence of pH on hydrogelator behavior.

### 2.8. Assessment of the Effect of Exposure to UV Light on Self-Assembly by Fluorescence Emission Spectroscopy

Hydrogelator 2 (1.0 mg) was dissolved in EtOH (5 mL) to afford a 0.40 mM solution. From this solution, 10 μL was taken and added to a solution of buffer (0.990 mL) at pH 5 (previously prepared from a sodium phosphate 0.1 M solution (0.1 M) and a mixed solution of citric acid (0.05 M) and boric acid (0.2 M), as described by Perrin and Dempsey) [45] to afford a solution of 4.0 μM. The emission spectrum was obtained for each hydrogelator solution to evaluate the influence of pH on hydrogelator behavior.

### 2.9. Scanning Transmission Electron Microscopy (STEM)

STEM images were recorded using a NanoSEM—FEI Nova 200 (FEI Technologies, Inc., Hillsboro, OR, USA), operating at 15 kV, coupled to an Electron Dispersive Spectroscopic analyzer (EDS) (FEI Technologies, Inc., Hillsboro, OR, USA) and Electron Backscatter Diffraction EDAX—Pegasus X4M analyzer (Pegasus Laboratories, Inc., Pensacola, FL, USA) and detection system (EBSD) at SEMAT (Serviços de Caracterização de Materiais), Guimarães, Portugal. After preparation of the hydrogel, a small portion of each sample was placed onto a TEM 400 mesh copper grid with Formvar/Carbon (ref. S162-4 from Agar Scientific Ltd, Essex, UK), held by tweezers and the excess solution was cleaned. The processing of STEM images was performed using ImageJ software (National Institutes of Health (NIH), Bethesda, MD, USA), which consisted in enhancing local contrast and adjusting brightness followed by manual selection of fibers.

### 2.10. Circular Dichroism Spectroscopy

The CD spectra were recorded at 20 °C using a spectropolarimeter Jasco model J-1500 spectropolarimeter (JASCO, Tokyo, Japan) at 25 °C under a constant flow of nitrogen gas. Peptide hydrogelator 0.01 wt.% solutions were loaded into 0.1 mm quartz cells. Spectra were acquired with 1 nm steps, 1 nm bandwidth, and 1 s collection time per step, taking three averages. The obtained data was smoothed by a 7-point Savitsky-Golay filter to remove random noise elements from the averaged spectra.

### 2.11. Cell Culture

Human keratinocytes (HaCaT cells) were from ATCC (Rockville, MD, USA). Cells were cultured in DMEM supplemented with 10% FBS and 1% penicillin/streptomycin, and were incubated at 37 °C, in a humidified atmosphere of 5% CO_2_.

### 2.12. DNA Quantification

HaCaT cells in the exponential growth phase were seeded and cultured (1.5 × 10^4^ cells/well) in 96-well plates and left to grow for 24 h. After 24 h incubation with the molecules under study, the culture medium was replaced by 50 µL of ultra-pure water, plates being incubated for 30 min at 37 °C and immediately frozen at –80 °C. DNA quantification was performed in a triplicate pool using a Qubit™ dsDNA HS Assay Kit according to manufacturer’s instructions. Results are expressed as percentage of the respective control and correspond to the mean ± SEM of three independent experiments performed in triplicate.

### 2.13. Wound Healing Assay

HaCaT cells were seeded and cultured in 2-well µ-chambers (2.0 × 10^4^ cells/well) with a separating wall in-between. When cells reached confluence (18–20 h after seeding), the media was removed and new media containing compound **3** (100 µM) was added for a 2-h pre-incubation period. After this step, the separating wall was removed and considered time-point zero. After this, cells were maintained in a desiccator and imaged after each hour of incubation.

Images were acquired in an inverted Eclipse Ts2R-FL (Nikon Instruments Inc., Melville, NY, USA) equipped with a Retiga R1 camera (QImaging, Surrey, BC, Canada) and an S Plan Fluor ELWD 10× DIC N1 objective (Nikon Instruments Inc., Melville, NY, USA). Images were analyzed with Fiji/ImageJ software version 1.51 (Fiji, Madison, WI, USA) [46]. Brightfield images were acquired, which were then transformed (Fiji’s “find edges” command + green LUT) in order to highlight the edges of the wound. For quantitative parameters, the width of the wound gap was measured using Fiji’s built-in measure function. For each experimental condition, at least 12 data points were recorded.

### 2.14. Statistical Analysis

For biological assays, the Shapiro–Wilk normality test was performed in the data to ensure that it followed a normal distribution. Comparison between the means of controls and each experimental condition was performed using ANOVA, except in the case of wound healing assay, in which Student’s t-test was used. Outliers were identified by the Grubbs’ test. Data was expressed as the mean ± SEM of at least 3 independent experiments, each performed in triplicate. GraphPad Prism software version 6.0 (GraphPad Prism Inc., San Diego, CA, USA) was used, and values were considered statistically significant with a *p* < 0.05.

### 2.15. Sustained Release Assays

Hydrogels of **2** were prepared as described in Section 2.2 in UV cuvettes, to form 1 mL hydrogels containing 4.0 mg of **2** and the appropriate cargo—methylene blue (0.1 mM), methyl orange (0.2 mM) or ciprofloxacin (0.2 mM), in a slightly modified version of the procedure described by Abraham et al [47]. After allowing to stand overnight, 1.5 mL of water was carefully added to the surface of the hydrogels, and the cuvettes were sealed. One set of hydrogels was irradiated with UV (360 nm) for 10 min after 3 h, and in this case aliquots (100 μL) of the upper liquid layer were taken at 1.5 h, 3 h, 6 h, 24 h, 48 h, 72 h, 96 h, and 168 h (after removing each aliquot, the volume was replaced with water). Another set of hydrogels was irradiated with UV (360 nm) at 168 h, and in this case aliquots (100 μL) were taken at 1.5 h, 3 h, 6 h, 24 h, 48 h, 72 h, 96 h, 168 h, 192 h, 216 h, and 240 h (after removing each aliquot the volume was replaced with water). The concentration of methylene blue or methyl orange in each aliquot, was determined by measuring the absorbance at λ_max_ of the dye (666 nm for methylene blue and 465 nm for methyl orange) using a microplate reader and then converting the value to a concentration (using a standard concentration curve) and then the percentage release. The concentration of ciprofloxacin in each aliquot was determined using analytical HPLC, where the integrated peak area was converted to a concentration (using a standard concentration curve) and then to the percentage release. Each experiment was performed in duplicate, and the mean percentage cargo release was plotted against time. The error is shown as the standard deviation about the mean.

## 3. Results

### 3.1. Chemical Synthesis of CNB-Phe-ΔPhe-OH (2) and H-Phe-ΔPhe-OH (3)

**Scheme 1:**
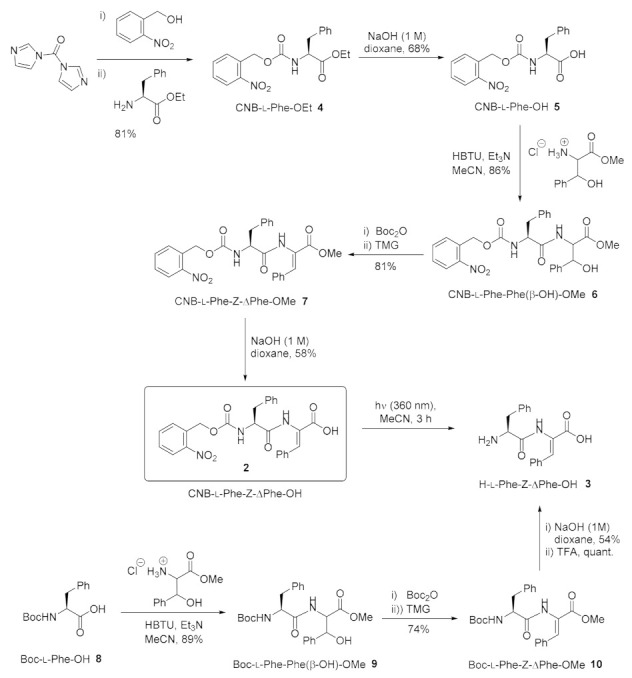
Synthesis of CNB-Phe-ΔPhe-OH (**2**) and H-Phe-ΔPhe-OH (**3**). Irradiation with UV light converts (**2**) to (**3**). The C=C double bond in dehydroamino acid residues is present as the Z geometric isomer.

The synthesis involved in this study is summarised is Scheme 1. The synthesis of CNB-Phe-ΔPhe-OH (**2**) was generally performed in a similar manner to our previously performed synthesis of Cbz-Phe-ΔPhe-OH (**1a**) [18], keeping all reactions and purifications away from direct light as much as possible to protect against premature loss of the photo-sensitive group. A key difference between the syntheses arose from the availability of the starting materials, as Cbz-Phe-OH is available commercially, whilst CNB-Phe-OH requires synthesis “in-house”. CNBCl was also not readily available. Therefore, we synthesized CNB-Phe-OEt (**4**) using a two-step procedure, employing two successive substitutions onto CDI, first with *o*-nitrobenzyl alcohol and then with H-Phe-Oet [48]. The resulting product, CNB-Phe-OEt (**4**) was saponified uneventfully with NaOH to afford CNB-Phe-OH (**5**).

Amide **6** was synthesized using a peptide coupling reaction between CNB-Phe-OH (**5**) and H-Phe(β-OH)-OH in the presence of HBTU. A subsequent dehydration reaction was performed utilizing a two-step procedure, where an initial attachment of a Boc group to the secondary alcohol was followed by a TMG-mediated elimination reaction, to afford **7**. Finally, hydrolysis of the methyl ester with NaOH gave the final product **2**. An initial test of the photo-reactivity of **2** was performed in MeCN using a UV lamp (RS-55 photochemical reactor). Irradiation at 360 nm for 3 h gave a clean conversion of **2** to dipeptide **3**, which was obtained in 96% yield following filtration. The identity of dipeptide **3** was corroborated via an independent synthesis, starting with a peptide coupling reaction between Boc-Phe-OH (**8**) and H-Phe(β-OH)-OH, to give **9**, which was dehydrated using the previously employed conditions (*vide supra*) to afford dehydrodipeptide **10**. Hydrolysis of the methyl ester, followed by removal of Boc group with TFA, gave the desired dipeptide **3** (Scheme 1). Carrying out these final two steps in reverse order gave a significant amount of the cyclic dipeptide, cyclo-(Phe-ΔPhe).

### 3.2. Gelation Study of CNB-Phe-ΔPhe-OH (**2**) and H-Phe-ΔPhe-OH (**3**)

The gelation experiments were conducted using 1 mL total gelation volume in small (5 mL) glass vials. CNB-Phe-ΔPhe-OH (**2**) showed limited solubility in buffer solutions in the physiological pH range (6–8), but could be rendered soluble upon the adjustment of water (1 mL) dispersions (0.3–0.6 wt.%; 3–6 mg/mL) to pH 10 with dilute NaOH (1 M, 20 μL). Gelation of **2** was triggered by slowly decreasing the pH to 5.5 by the addition of GdL (0.3 wt.%; 3 mg/mL). The solutions were left to stand undisturbed overnight at this point to stabilize. Gelation was observed, and the minimal concentration required for peptide gelation (the critical gelation concentration, CGC), was determined for CNB-Phe-ΔPhe-OH (**2**) as 0.4 wt.% (4 mg/mL) via inverted tube tests. This value is higher than, but nonetheless the same order of magnitude as, the previously studied related hydrogels **1a** and **1b** (0.1 and 0.2 wt.% CGCs, respectively) [12]. The higher CGC observed for compound **2** possibly reflects a disruption of the π-stacking by the additional nitro group. In that same previous study, **1a** was able to also form a hydrogel at pH 7.3 via a heating–cooling cycle. Dipeptide **2** did not form a hydrogel under these conditions, in the concentration range of 0.3-1.0 wt.% (3–10 mg/mL). Again, this might be explained by a reduced π-stacking ability of the CNB group compared to Cbz group.

As mentioned previously, H-Phe-ΔPhe-OH (**3**), the photo-deprotection product of **2**, has been previously reported as being able to form a hydrogel using a solvent switch method (hexafluoroisopropanol to 0.8 M NaOAc solution). We were keen to see if **3** was also to form a hydrogel using our gelation conditions based on a pH change, as this might give a clue as to whether our system would be photo-responsive. Thus, hydrogelation was attempted using the aforementioned conditions of adjusting samples (0.3–1.5 wt.%, 3-15 mg/mL) to pH 10 with NaOH (1 M) and then slowly reducing the pH with GdL. After standing undisturbed overnight, no hydrogelation occurred (pH dropped to 5.6). In all cases, no precipitate was observed, so it was concluded that **3** is too soluble in these aqueous conditions for gelation to occur (the self-assembly process also suffers from a reduced number of π-stacking units), at least under the conditions studied. Taken together, these gelation studies suggested that the photo-deprotection of gels consisting of CNB-Phe-ΔPhe-OH (**2**) to H-Phe-ΔPhe-OH (**3**) may be able to provide a gel-to-solution, or gel-to-sol, transition.

### 3.3. Visual Study of Gel-to-Sol Transition of CNB-Phe-ΔPhe-OH (2)

**Figure 2:**
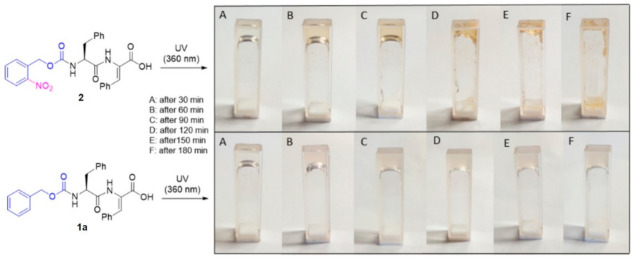
Inverted tube test of a 0.4 wt.% hydrogel of **2** (top panel) and **1a** (bottom panel) after irradiation with UV light (360 nm) for 30 (**A**), 60 (**B**), 90 (**C**), 120 (**D**), 150 (**E**) and 180 (**F**) minutes—photographs taken 5 min after inversion.

It was envisaged that exposing CNB-Phe-ΔPhe-OH (**2**) to UV light would generate the more water-soluble dipeptide H-Phe-ΔPhe-OH (**3**), accompanied by a disruption to the hydrogel fiber network. Therefore, a gel composed of **2** might be expected to undergo a gel-to-sol transition upon exposure to UV light, along with precipitation of the aqueous-insoluble *o*-nitrosobenzaldehyde by-product. To this end, a hydrogel of **2** was prepared in a UV cuvette in the same way as described in Section 2.2 (0.4 wt.%, 4.0 mg/mL) and left to stabilize overnight. The sample of hydrogel was then irradiated with UV light (360 nm) for 3 h, with an inverted tube test being made every 30 min. We were concerned that repeated inversions might have a destabilizing effect on the gel, and therefore a gel of Cbz-Phe-ΔPhe-OH (**1a**) (not photo-labile) was used as control, to verify that any gel-to-sol transition could be attributed solely to the removal of the aromatic capping group. After irradiating **2** with UV (360 nm) for 1 h, a slight darkening was observed, and a small amount of water was extruded from the gel (Figure 2, top, B). After 2 h, the onset of gel disintegration was observed (Figure 2, top, D), and after 3 h the gel-to-sol transition was complete (Figure 2, top, F). In contrast, irradiation of **1a** for 3 h produced no clear visible change to the gel composition (Figure 2, bottom, A–F). 

In the HPLC study reported in Section 3.4 (*vide infra*), a t_1/2_ of ~20 min was observed for the photo-cleavage of **2**. This suggested a considerable lag-time between the photochemical reaction occurring and the disruption of the hydrogel network manifesting into the dissolution of the gel, as a much shorter UV exposure time would be expected to lower the concentration of **2** to below the CGC. Considering a possible lag-time between protecting group removal and gel dissolution, we investigated shorter UV exposure times, this time in the presence of a simulated biological fluid (SBF, prepared according to a literature procedure) in order to better mimic a biological environment [49]. Three hydrogels were prepared, before SBF was carefully layered on the top of the gels. One was irradiated with UV light (360 nm) for 20 min, one for 10 min and one was not exposed to UV light. Interestingly, after 3 h, we observed that the gel which was irradiated for 20 min produced an almost homogeneous single liquid phase, whereas the gels irradiated for 10 min and 0 min were still visibly intact (see Supplementary Information). This result suggests a threshold degree of photo-reaction is required to afford gel dissolution,

### 3.4. HPLC Study of the Time-Course of CNB-Phe-ΔPhe-OH (**2**) Conversion to H-Phe-ΔPhe-OH (**3**) under UV Light (360 nm) Exposure

**Figure 3:**
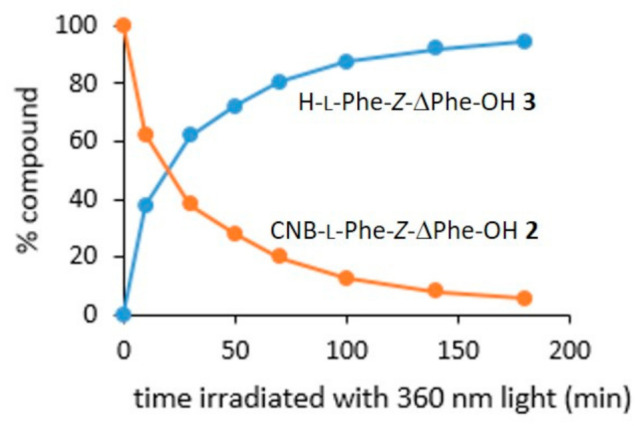
Conversion of compound **2** to compound **3**, under UV light (360 nm), followed by HPLC (10, 30, 50, 70, 100, 140, and 180 min irradiation). Percentages were adjusted to allow for the differences in relative response factor, using a calibration graph obtained from the analysis of authentic samples of compounds **2** and **3** (see Supporting Information).

In order to study the gel-to-sol transition described in Section 2.3 more quantitatively, the experiment was repeated and followed by HPLC. To this end, GdL (0.3 wt.%, 3 mg/mL) was added to a sample of CNB-Phe-ΔPhe-OH (**2**) (0.4 wt.%, 4 mg/mL) in a solution of water (1 mL) and NaOH solution (1 M, 20 μL) in a UV cuvette, before being allowed to stabilize overnight in the dark. The sample in the cuvette was then irradiated with UV light (360 nm) for 3 h, and small portions from various positions from within the hydrogel/sol (3 × 10 μL) were taken at 10, 30, 50, 70, 100, 140, and 180 min. The samples were diluted with MeCN (750 μL) and analyzed by HPLC. Through a comparison with authentic samples, HPLC analysis showed the conversion of CNB-Phe-ΔPhe-OH (**2**) to H-Phe-ΔPhe-OH (**3**), and plotting this conversion against time revealed an initial t_1/2_ of ~20 min (Figure 3). The t_1/2_ increased slightly as the reaction progressed, and this decrease in reaction rate is possibly due to the UV-protective effect of the released aromatic deprotection product. After 3 h, the conversion was >95% complete. The HPLC traces also showed two additional peaks, which could be attributed to GdL and its hydrolysis product, confirmed by comparison with a reference sample (example HPLC traces are shown in the Supporting Information).

### 3.5. ^1^H NMR Study of the Time-Course of CNB-Phe-ΔPhe-OH (**2**) Conversion to H-Phe-ΔPhe-OH (**3**) under UV Light (360 nm) Exposure

The conversion of CNB-Phe-ΔPhe-OH (**2**) to H-Phe-ΔPhe-OH (**3**) upon exposure to UV light (360 nm) was studied by ^1^H NMR, under a diluted version of the final gel conditions, within an NMR tube. The hydogelation conditions presented in Section 2.3 were modified slightly, in that **2** was dissolved in a mixture of D_2_O and NaOD (1 M) solution, instead of H_2_O and NaOH (1 M) solution, to a final concentration of 0.3 wt.%. After the addition of GdL, the solution was immediately transferred from the vial to an NMR tube and the solution was left overnight to allow self-assembly to occur. The ^1^H NMR spectrum of the resulting sample showed the expected resonances for CNB-Phe-ΔPhe-OH (**2**) and for GdL. 

**Figure 4:**
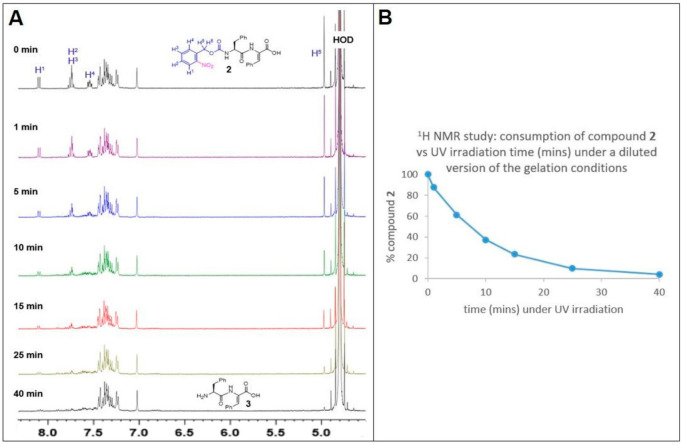
Panel (**A**): ^1^H NMR spectra showing the removal of the carboxy-2-nitrobenzyl (CNB) group, to form **3** from **2**, at different time-points following irradiation with UV light (360 nm), after performing a diluted version the gelation in an NMR tube. Panel (**B**): The percentage of CNB group removal at each time-point was calculated by integration, and the percentage conversion was plotted against the time irradiated with 360 nm UV light (mins).

As expected, CNB-Phe-ΔPhe-OH (**2**) converts to H-Phe-ΔPhe-OH (**3**) upon UV exposure, evidenced by the observed loss of the four aromatic protons and two benzylic protons assigned to the CNB group (Figure 4A). The other proton resonances in the spectrum are essentially unchanged, as expected. In the conversion time-course (Figure 4B), **2** has a half-life of ~8 min, and after 40 min the conversion is >95% complete. The *o*-nitrosobenzaldehyde by-product, which is water insoluble and therefore is invisible by ^1^H NMR under the study conditions, was visible as a precipitate at the bottom of the NMR tube. The time-course of conversion observed here is faster than the conversion rate observed by HPLC in Section 2.4 (t_1/2_ = 8 min vs. t_1/2_ = 20 min). This can be explained by considering the different dimensions of the UV cuvette and the NMR tube. The UV cuvette used in Section 2.2 and Section 2.3 is wider, providing an increased inner-filter effect and an increased protection from the UV light. When the study is conducted in an NMR tube, the narrower dimensions and greater surface area of the vessel provide a greater overall exposure of the sample to the UV light.

### 3.6. Rheological Study of CNB-Phe-ΔPhe-OH (**2**) and Comparison with Cbz-Phe-ΔPhe-OH (**1a**)

**Figure 5:**
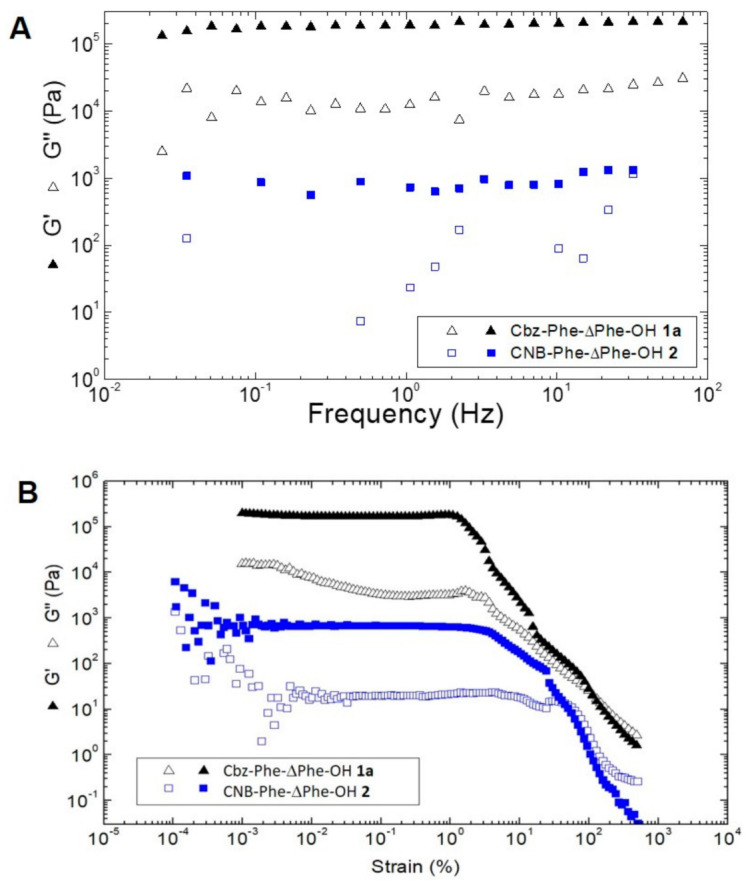
(**A**): Frequency dependence of the shear elastic G′ (empty symbols) and loss G′′ (filled symbols) moduli for hydrogels formulated at 0.4 wt.% of hydrogelator **2** (squares) and **1a** (triangles). (**B**): Strain dependence of the shear elastic G’ and loss G’’ moduli for hydrogels formulated at 0.4 wt.% of hydrogelator **1a** (black) and **2** (blue).

The mechanical spectra of equilibrated hydrogels demonstrates that the gel of **2** is frequency-independent (Figure 5A). The effects of the structural chemical modification on hydrogel elasticity are underlined in Figure 5B. Hydrogels consisting of **2** show a two orders of magnitude decrease of the shear elastic modulus, compared with the parent compound **1a**, which lacks the nitro group on the capping unit [12]. This reduced gel strength could be due to the extra nitro group disturbing the π-stacking interactions. In fact, gels consisting of **2** behave more like gels of **1b** (Cbz-Tyr-ΔPhe-OH), which might be expected as **2** and **1b** both contain the same number of hydrogen bond acceptors. The similarity in gelation structure between **2** and **1b** is also apparent in the STEM images (*vide infra*).

If the hydrogels are assumed to be incompressible, the shear modulus G is of the same order of magnitude as G′, and G′ ≈ G = E/3 [14]. Despite **2** being a weaker gel than **1a**, the elastic modulus of **2** still falls in the range of the native tissues, 0.1 kPa (brain) to 100 kPa (cartilage), that covers soft tissues such as skin, pancreas, spleen, glands, and muscles [50]. Therefore, from a mechanical perspective, the elastic properties at 0.4 wt.% are suitable for biomedical applications.

A comparison of the rheology results for **2** and **1a** is perhaps at odds with the STEM microscopy results (*vide infra*, Section 3.8), where more fibers are observed for a less stiff gel. If one assumes that the Flory theory for rubber elasticity applies to the peptide gels studied here, one may relate the gel elastic modulus G, measured in the mechanical spectra from the value of the constant storage modulus G’, with the crosslink density N (number of network strand per unit volume) using the relationship [51] G = NkT, where T is the temperature and k the Boltzmann constant].

It is evident that this trend is opposite to the one suggested by the gel structures presented in Section 3.8, since more fibers are imaged for gel **2** than for gel **1a**. In the Flory theory, the elasticity of the network originates from the change in the conformational entropy of flexible polymer strands resulting from the network deformation. However, such contribution is known to be negligibly small in networks made of rod-like strands where elasticity is mainly due to the deformation of the individual strand and thus essentially depends on the elastic properties of the strand and its rod-like character [52]. Thus, one cannot simply connect the gel elasticity to the mesh size or fiber density imaged in Figure 7. Instead, a lower bending modulus of less rigid-like fibers, a larger number of elastically non effective fibers (and thus a drop in N as such fibers are not network strands) or even non affine network deformation can be invoked, among other factors, to explain the lower modulus G exhibited by gel **2**.

### 3.7. Effect of pH on Self-Assembly of CNB-Phe-ΔPhe-OH (**2**) and Comparison with Cbz-Phe-ΔPhe-OH (**1a**)

Fluorescence spectroscopy was used to assess the effect of pH on the self-assembly of hydrogelator **2**, performed at a concentration below the critical gelation concentration (CGC), but above the critical aggregation concentration (CAC) (4.0 μM). As the pH of the solution of the deprotonated carboxylate is decreased from 10 towards the pKa of the hydrogelator, the anionic species starts to become protonated to the neutral form, creating a balance of repulsive and attractive forces, leading to self-assembly. The self-assembly process is accompanied by conformational changes, which in turn result in changes to the fluorescence emission spectrum.

**Figure 6:**
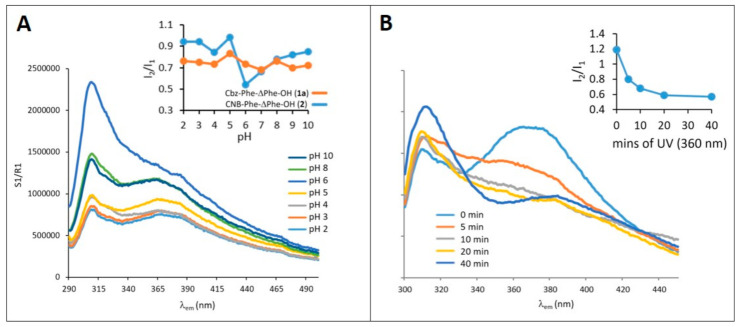
Panel (**A**): Dependence of fluorescence emission on pH of hydrogelator **2** (10 µM, λ_exc_ = 280 nm). Panel (**A**) inset: pH dependence of aggregate (I_2_, measured at 370 nm) to monomer (I_1_, measured 315 nm) emission band intensity ratio of hydrogelator **2** and comparison with **1a**. Panel (**B**): Change of fluorescence emission of hydrogelator **2** following UV irradiation for 0 min, 5 min, 10 min, 20 min, and 40 min (measured at pH 5). Right: Fluorescence emission spectrum of hydrogelator **2** at pH 5 versus time irradiated with 360 nm UV light (mins). Panel (**B**) inset: Aggregate (I_2_, measured at 370 nm) to monomer (I_1_, measured 315 nm) emission band intensity ratio of hydrogelator **2** versus time irradiated with 360 nm UV light (mins).

The fluorescence emission spectra of hydrogelator **2** shows a main band with maxima at ~315 nm, and a second fluorescence emission band with maxima at ~370 nm (Figure 6A), which are associated with monomeric and aggregated species, respectively [13,14,15,16]. The aggregate-to-monomer ratio (I_2_/I_1_) was plotted against pH (Figure 6A, inset). Hydrogelator **2** follows a trend similar to hydrogelator **1a** (and other reported hydrogelators) [12], where an increase of the aggregate-to-monomer emission ratio is observed when pH of the gelator solution is slightly above the pKa of the carboxylic acid (expected pKa ~4), with an optimal pH of pH 5. This fits closely with the hydrogelation results in Section 3.2, where gelation of **2** occurred at pH 5.5. As the pH is further reduced to pH 2, the aggregate-to-monomer ratio decreases. Above pH 5, the aggregate-to-monomer ratio drops for both **2** and **1a**, sharply in the case of **2**. The level of aggregation recovers somewhat in both cases when pH > 7. The profile for **1a** then shows a secondary maximum at pH 8. This, and the overall shallower profile, may explain why **1a** forms a hydrogel at pH 7.3 with a heating/cooling cycle, whereas **2** does not.

It was envisaged that the band observed for aggregates at 370 nm would decrease upon exposure to UV light (360 nm), due to the degradation of the aggregating species. A solution of **2** at the gelation pH (pH 5.5) was irradiated with UV light (360 nm) and a fluorescent spectrum was recorded at 5, 10, 20, and 40 min (Figure 6B). Plotting the I_2_/I_1_ ratio versus time showed that aggregates were converted to monomer over time, as would be expected if the self-assembling species were being consumed (Figure 6B, inset).

### 3.8. Studying the Nano/Micro Structure of CNB-Phe-ΔPhe-OH (**2**) Hydrogel by STEM

**Figure 7:**
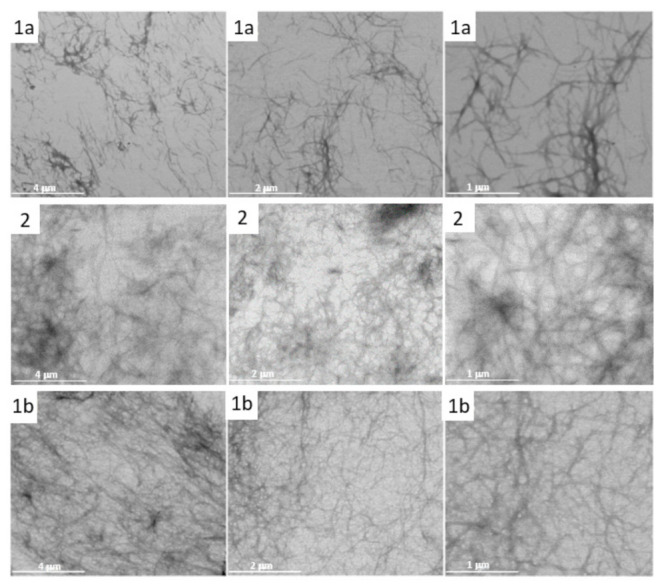
Comparison of scanning transmission electron microscopy (STEM) images of hydrogels **1a** (top), **2** (centre), and **1b** at 0.4 wt.%, at different magnifications.

The nano/microstructure of the three-dimensional hydrogel fibers was investigated using scanning electron microscopy in transmission mode (STEM). STEM images of hydrogel **2**, prepared at 0.4 wt.%, were obtained and compared with those for the previously studied **1a** and **1b** (Figure 7) [12]. Hydrogel **2** displays a densely packed fibrous structure comprising of thin tangled fibers. The fibrous structure of hydrogel **1a** is less densely packed than for hydrogel **2**, but the dimensions of the individual tangled fibers are similar, with average fiber cross-sections of 40.0 ± 9.0 nm (for **2**) versus cross-sections of 26.1 ± 4.6 nm (for **1a**) and 34.5 ± 6.8 nm (for **1b**). The density of the fibers for hydrogel **2** are in fact more similar to Cbz-Tyr-ΔPhe-OH (**1b**) than Cbz-Phe-ΔPhe-OH (**1a**), which is perhaps expected as the extra hydroxyl of the tyrosine residue means that **1b** has the same number of hydrogen bond acceptors as **2**, whereas **1a** has one fewer hydrogen bond acceptors than **2**. However, it is not readily apparent why **1a**, which shows less dense fibers, has a lower CGC value and stronger rheological properties, than **2** and **1b**.

### 3.9. Studying the UV-Promoted Degradation the CNB-Phe-ΔPhe-OH (**2**) from Cotton Gauze Wound Dressing by STEM

**Figure 8:**
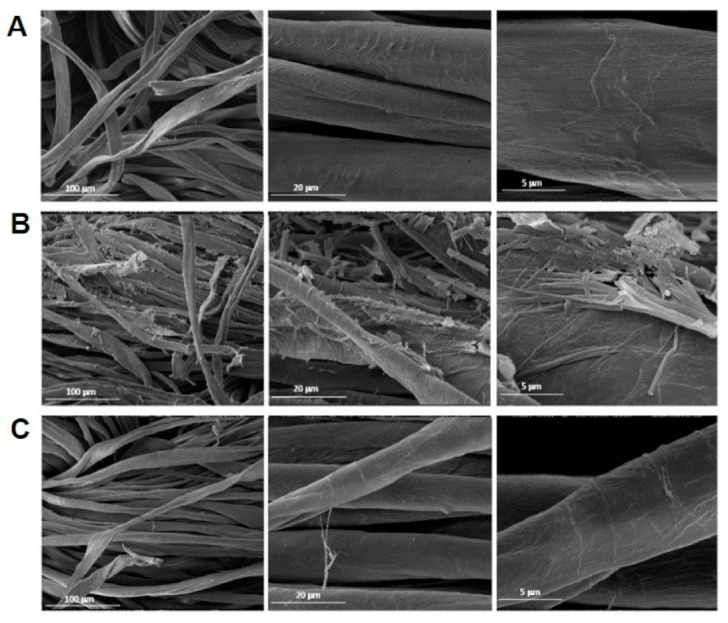
STEM images of a hydrogel formed within cotton gauze wound dressing. (**A**): Control, cotton dressing submerged in NaOH/GdL solution (without hydrogelator 2, without UV exposure). (**B**): Hydrogel formed within the cotton dressing, by submerging in hydrogelator 2/NaOH/GdL solution (without UV exposure). (**C**): Cotton dressing submerged in hydrogelator 2/NaOH/GdL solution, and then exposed to UV light (360 nm, 1 h).

In order to study the behavior of the hydrogel within the cotton fibers of a model cotton gauze dressing, we first prepared the pre-gelation mixture of **2** in a glass vial, as described in Section 2.2. After the addition of GdL and mixing, a small square of cotton gauze wound dressing (1 cm × 1 cm) was submerged in the solution for 1 min and then removed using tweezers and placed inside a sealed eppendorf. The gelation process was allowed to develop undisturbed within the cotton fibers overnight. The piece of cotton wound dressing was then exposed to UV light (360 nm) for 1 h and analyzed by STEM (Figure 8C). For comparison, the same process was repeated without UV irradiation of the sample (Figure 8B). As an additional control, the hydrogelator **2** was omitted from the preparation, so that the cotton square was submerged in a solution containing just dilute NaOH and GdL (Figure 8A). These control experiments would allow us to be certain which fibers observed by STEM could be assigned to the structure of the cotton and which could be assigned to a formed hydrogel.

The STEM results show that when the hydrogel is formed within the cotton gauze dressing (and not exposed to UV), extra fibers are visible, which are attached to the larger fibers of the cotton (Figure 8B). These fibers were not observed when the hydrogelator itself was omitted from the preparation, in the control sample, confirming that the observed extra fibers are formed of hydrogel (Figure 8A).

When the same preparation of hydrogel within the cotton structure was performed and then subsequently exposed to UV light (360 nm) for 1 h, analysis by STEM indicated that these observed extra fibers had been dissolved from the cotton fiber structure (Figure 8C). This result using this model UV-responsive hydrogel shows that there may be potential for releasing biologically active dehydropeptides from within a wound dressing, when required.

### 3.10. Studying the Secondary Structure of CNB-Phe-ΔPhe-OH (**2**) and H-Phe-ΔPhe-OH (**3**) by Circular Dichroism Spectroscopy

**Figure 9:**
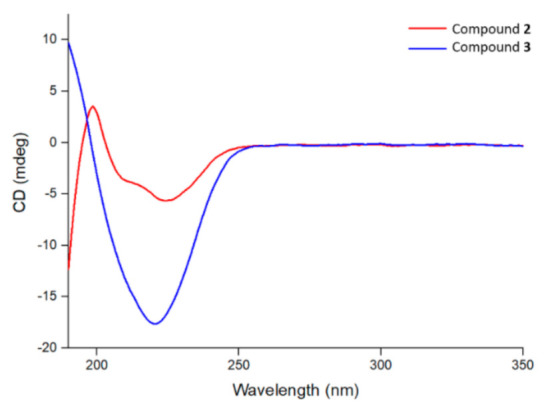
Circular dichroism spectra of compounds **2** and **3** at 0.01 wt.%.

The secondary structures of **2** and **3** were studied using circular dichroism spectroscopy (Figure 9) [53]. The spectra were acquired under a diluted version of the gelation conditions (0.01% wt.%) to avoid the scattering affects which can occur in turbid gels. Although the hydrogelator concentration is below the CGC, it is likely that the hydrogelator concentration is above the critical aggregation concentration (CAC), and therefore similar structures to the hydrogel will exist in solution. The CD spectrum of compound **2** most closely resembles that of an α-helix, although the positive band at 200 nm suggests there may also be some β-sheet character present. The large negative band in the CD spectrum of compound **3** suggests a β-sheet secondary structure, although the spectrum lacks a positive band at around ~195 nm. The spectrum of **3** is similar in appearance to that reported by Chauhan for the same compound [38].

### 3.11. Biocompatibility

**Figure 10:**
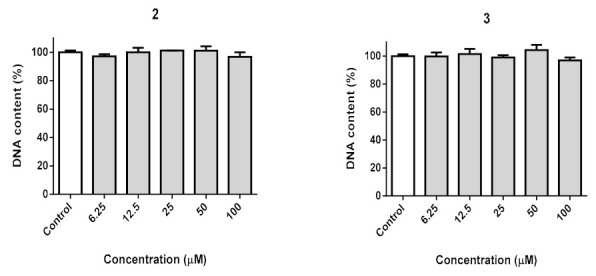
DNA content of HaCaT cells treated with CNB-Phe-ΔPhe-OH (**2**) and H-Phe-ΔPhe-OH (**3**) for 24 h, at the concentrations presented.

The compounds of CNB-Phe-ΔPhe-OH (**2**) and its photo-degradation product, H-Phe-ΔPhe-OH (**3**), were evaluated for their toxicity to human cells. More specifically, the putative impact of the molecules upon the cell proliferation of the human keratinocyte cell line, HaCaT, was assessed. To this end, we evaluated the DNA content of treated cells when compared to control conditions. As seen in Figure 10, no impact in cell proliferation was observed with both compounds **2** and **3**.

Even though this photo-responsive system reported here is intended as a model system, we did conduct one further biological study with the photo-released compound, H-Phe-ΔPhe-OH (**3**), related to a skin application. To evaluate the potential impact in cell growth in a context of wound healing, we have used a scratch assay. In our model (see Supplementary Figure S6), cells were allowed to grow in two different µ-chambers until confluence, after which the division between the chambers was removed, with full closure being attained after approximately 6 h.

**Figure 11:**
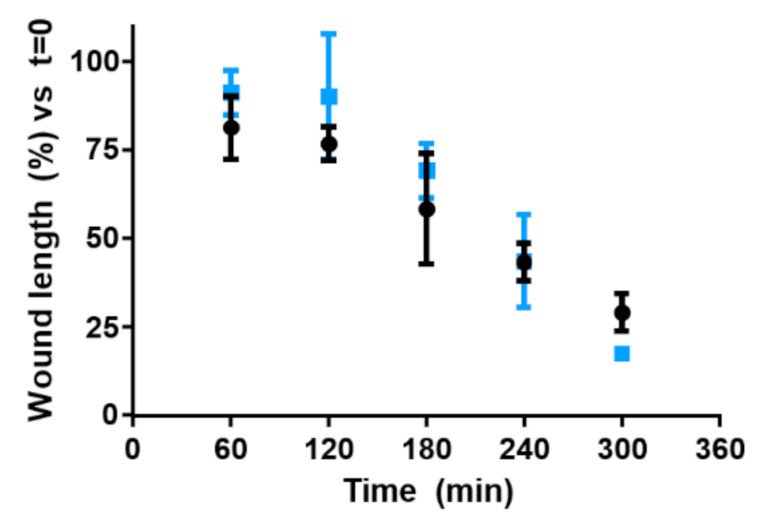
Width of wound gap though time, when compared with t = 0 (maximum width). Black series: control; Blue series: cells incubated with H-Phe-ΔPhe-OH (**3**) at 100 µM.

As shown in Figure 11, H-Phe-ΔPhe-OH (**3**) initially had no impact on the rate at which the wound gap closes, being indistinct from control cells up to 4 h after the beginning of the experiment. A difference is observed at the last time-point recorded, specifically after 5 h of incubation, with cells treated with H-Phe-ΔPhe-OH (**3**) displaying smaller gaps when compared with controls.

### 3.12. In Vitro Drug Delivery Studies

Drug delivery simulation studies were carried on gels of CNB-Phe-ΔPhe-OH (**2**), to determine if a short treatment with UV light could stimulate an increased rate of release of model drug compound from within a disrupted, or partially disrupted, gel matrix. Following an adaptation of a method described by Abraham et al [47], the dyes methylene blue and methyl orange were used as examples of negatively and positively charged model drug compounds, respectively, which after incorporation into hydrogels their release could be studied by measuring the absorbance at their respective λ_max_ values of 666 nm and 486 nm. The antimicrobial compound, ciprofloxacin, was also used, as an example of an overall neutral compound, whose release could be studied by HPLC [54]. Briefly, in all three cases, a 20-min UV irradiation (360 nm) provided a complete gel dissolution and ultimately the near complete release of drug cargo. Interestingly, a 10-min UV irradiation left the gel structures intact, while providing an increase in the rate of drug cargo release, versus control. Therefore, a 10 min UV irradiation time represents an intermediate timeframe where the drug cargo can be mobilized without destroying the gel structure, potentially allowing tunable drug release. A full description and figures are provided in the Supporting Information.

## 4. Discussion

The initial aim of this work was to synthesize CNB-Phe-DPhe-OH (**2**) and then to investigate whether it could form a hydrogel, and if so, to investigate a possible UV-responsive gel to liquid transition.

We were able to successfully synthesize CNB-Phe-ΔPhe-OH (**2**) in five steps using a readily scalable route. The compound was able to form a hydrogel with a critical gelation concentration of 0.4% and a pH of 5.5. The pH of the hydrogel is lower than that of biological systems in general, but has relevance for skin, duodenum, jejunum, and reproductive organ applications.

STEM microscopy images of gels of **2** revealed a microstructure of fibrils similar to related non-nitrated compounds, particularly compound **1b**. Rheological experiments showed that **2** is a weaker gel than its non-nitrated analogue, possessing an elasticity of more than order of magnitude less than that of **1a**. The lower gel strength (and higher CGC) of **2** compared with **1a**, whilst usually undesired, might in this case allow a more facile gel to solution transition.

UV irradiation experiments showed that **2** could be photolytically cleaved with UV light (360 nm) to dipeptide **3**, in organic solvent in the first instance, and then later under the gelation conditions. When a gel formed at 0.4% in a cuvette was irradiated with UV light (360 nm), the gel could visibly be seen to dissolve, whereas the non-nitrated version stayed as a gel. This UV-responsive gel to liquid transition was tested for its ability to accelerate in vitro drug release. This study revealed that a 20-min irradiation with UV light (360 nm) provided a complete cargo release of the antimicrobial, ciprofloxacin, and other model drug compounds, on-demand, accompanied by complete gel dissolution within 24 h. A 10-min exposure to UV light, on the other hand, afforded a small-to-modest increase in the rate of drug release relative to a non-irradiated system, this time leaving the hydrogel visibly intact, even after 10 days.

Hydrogels in general show attractive properties as wound dressing materials. Photo-responsive versions have previously found applications in wound dressings for the on-demand release of antimicrobials in response to infection, and for dissolution-on-demand materials to facilitate pain-less dressing removal [25,26] With this in mind, we conducted a preliminary investigation using STEM microscopy, to study a gel of **2** prepared on the surface of cotton gauze dressing. The gel nanostructure could be seen to dissolve from within the cotton fibers upon exposure to UV light (360 nm). The dehydropeptide photolysis product **3** showed a mild skin healing property in an in vitro scratch assay, whilst both **2** and **3** are non-toxic to a human keratinocyte cell line, HaCaT. Taken together, the compounds are worthy of further investigation in a wound healing context in vivo.

## 5. Conclusions

We have shown that CNB-Phe-ΔPhe-OH (**2**) behaves as a stimuli-responsive hydrogel, undergoing a gel to solution transition in response to UV light (360 nm), and releasing compound **3**. This model system is complementary to similar previously reported systems, in this case generating a more proteolytically stable dehydrodipeptide. The future work will involve in vivo studies relating to specialized applications, as well as adapting the system towards the “self-delivery” of biologically active dehydrodipeptides known in the literature [36,37,38,39,40].

## Data Availability

Data sharing not applicable.

## Supplementary Materials

The following are available online at https://www.mdpi.com/2079-4991/11/3/704/s1, Figure S1: ^1^H NMR (400 MHz, d_6_-DMSO), ^13^C NMR (100 MHz, d_6_-DMSO) and DEPT NMR (100 MHz, d_6_-DMSO) spectra for compound **2**, Figure S2: ^1^H NMR (400 MHz, d_6_-DMSO), ^13^C NMR (100 MHz, d_6_-DMSO) and DEPT NMR (100 MHz, d_6_-DMSO) spectra for compound **3**, Figure S3: Visual study of gel-to-sol transition of CNB-Phe-ΔPhe-OH hydrogel in contact with SBF, Figure S4: Photo-catalysed formation of **3** from **2**, the UV-promoted conversion of compound **2** into compound **3** followed by HPLC, calibration graph (for determining relative response factors) for compounds **2** and **3**. Figure S5: HPLC traces showing the conversion of compound **2** to compound **3**. Traces for reference samples of **2**, **3** and GdL are also shown, Figure S6: (**A**) Brightfield mosaic of several fields obtained with the 10x objective. (**B**) Processed images, resulting from the transformation of raw images with the software’s built-in “edge” macro and green LUT. (**C**) Width of wound gap though time, when compared with t = 0 (maximum width). Black series: control; blue series: cells incubated with H-L-Phe-Phe-OH **3** at 100 µM, Figure S7: (**A**) Graph showing percentage release of methylene blue from hydrogel of 2 when irradiated with UV light (360 nm) for 10 minutes after 168 hours (light blue line) or after 3 hours (dark blue line). (**B**) Photographs showing the appearance of the methylene blue hydrogel experiment at the start and the end of the experiment. (**C**) Graph showing percentage release of methyl orange from hydrogel of 2 when irradiated with UV light (360 nm) for 10 minutes after 168 hours (light orange line) or after 3 hours (dark orange line). (**D**) Photographs showing the appearance of the methyl orange hydrogel experiment at the start and the end of the experiment. (**E**) Graph showing percentage release of ciprofloxacin from a hydrogel of 2 when irradiated with UV (360 nm) for 10 minutes after 3 hours (light grey line) or without any UV irradiation (dark grey line). (**F**) Photographs showing the appearance of the ciprofloxacin hydrogel experiment at the start and the end of the experiment.

## Abbreviations

εAhx: 6-aminohexacosanoate; All, allyl; CAC, critical aggregation concentration; Cbz, carboxybenzyl or benzyloxycarbonyl; CD, circular dichroism; CGC, critical gelation concentration; CNB, carboxy-2-nitrobenzyl; ECM, extracellular matrix; GA, glucosamine; GdL, glucono-delta-lactone; HBTU, hexafluorophosphate benzotriazole tetramethyluronium; Npx, naproxen; NSAID, non-steroidal anti-inflammatory drug; TFA, trifluoroacetic acid; TMG, 1,1,2,2-tetramethylguanidine; STEM, scanning transmission electron microscopy; SDF, simulated body fluid.
