# Supplementary material for: Evaluation of a Model Photo-Caged Dehydropeptide as a Stimuli-Responsive Supramolecular Hydrogel
Source: Nanomaterials (Basel). 2021 Mar 11;11(3):704. doi: 10.3390/nano11030704 (PMC8001155; doi:10.3390/nano11030704)
Supplement: Supplementary file 1 [file nanomaterials-11-00704-s001.pdf]

Supplementary Information

for

Evaluation of a Model Photo-Caged Dehydropeptide as a Stimuli-rResponsive Supermolecular Hydrogel

Peter J. Jervis, José A. Martins, Loic Hilliou, Renato B. Pereira, David M. Pereira, Paula M. T. Ferreira

Contents

Page 1 NMR spectra for compound **2**

Page 2 NMR spectra for compound **3**

Page 3-5 Synthetic chemistry

Page 6 UV-promoted hydrogel dissolution study in contact with simulated biological fluid (SBF) figure

Page 7 Supplementary HPLC data for the UV-promoted conversion of compound **2** into compound **3**

Page 8 Supplementary data related to scratch assay wound healing model

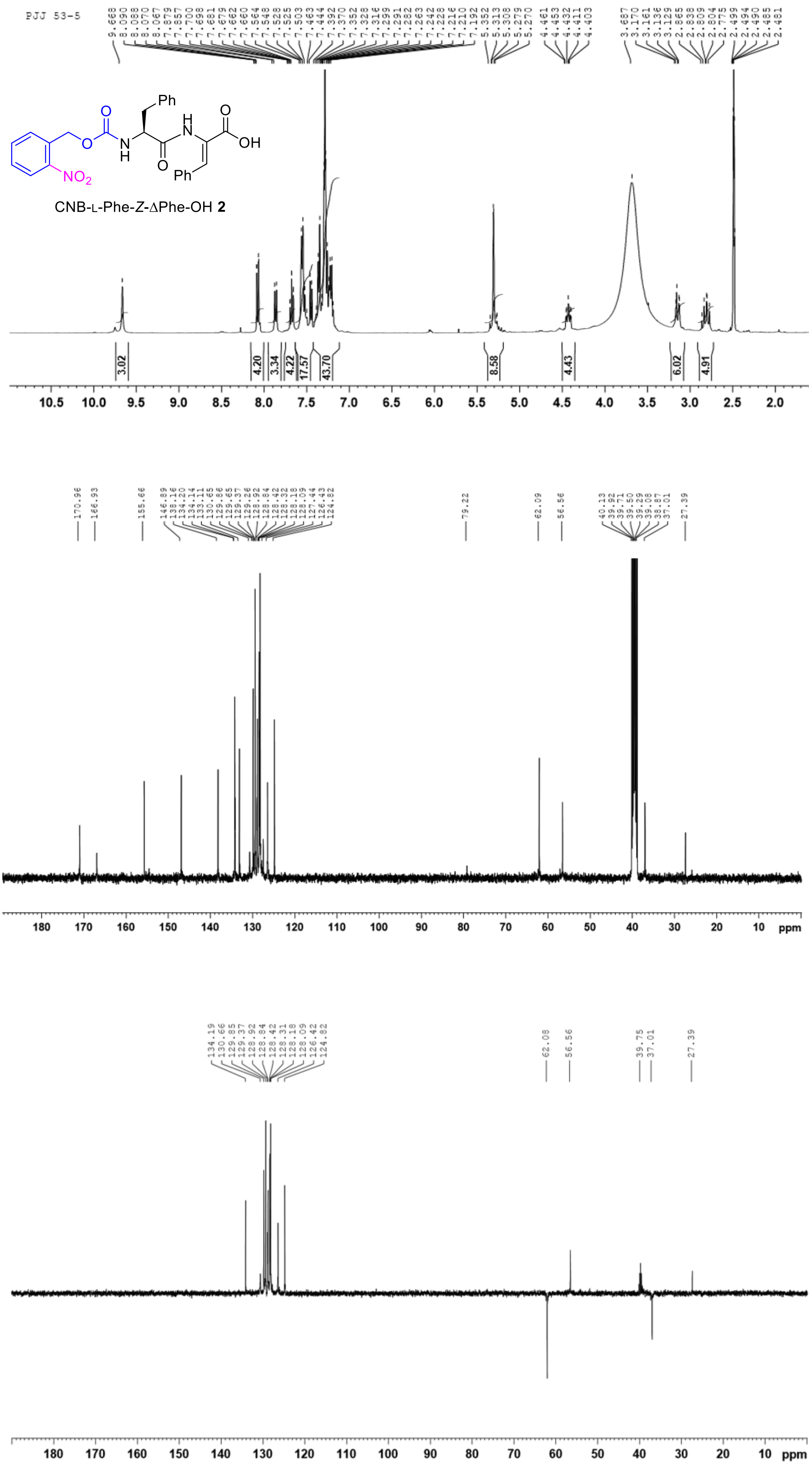

Figure S1: ^{1}H NMR (400 MHz, d$_6$-DMSO), ^{13}C NMR (100 MHz, d$_6$-DMSO) and DEPT NMR (100 MHz, d$_6$-DMSO) spectra for compound **2**.

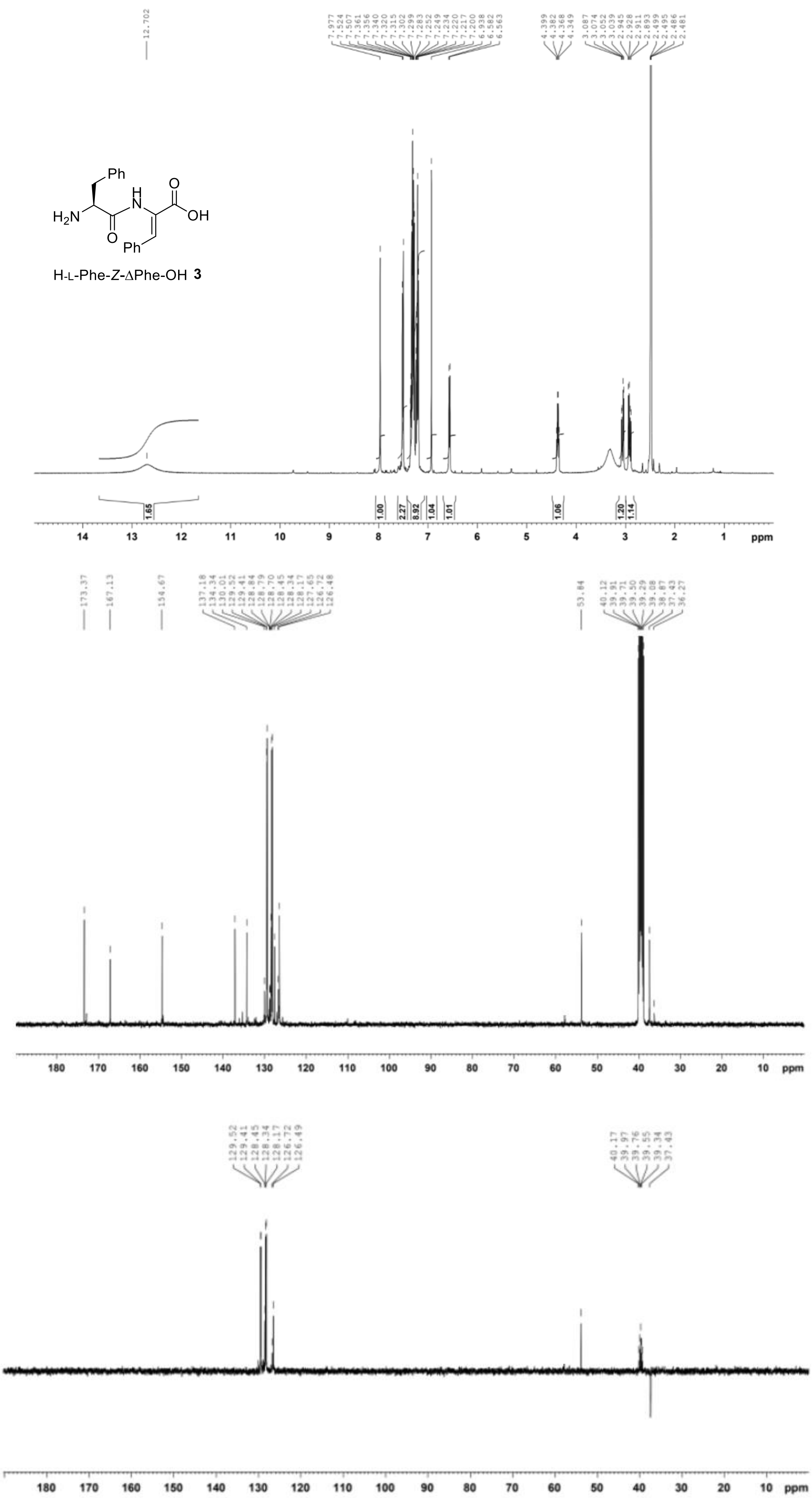

Figure S2: ^{1}H NMR (400 MHz, d_6-DMSO), ^{13}C NMR (100 MHz, d_6-DMSO) and DEPT NMR (100 MHz, d_6-DMSO) spectra for compound **3**.

Synthetic Chemistry

^{1}H and ^{13}C NMR spectra were recorded on a Bruker Avance III at 400 and 100.6 MHz, respectively. DEPT θ 45° and 135°, HMQC and HMBC were used to attribute some signals. Chemical shifts (δ) are reported in parts per million (ppm) and coupling constants (J) are reported in Hertz (Hz). Petroleum ether refers to the boiling range of 40-60 °C. Acetonitrile was dried over silica and calcium hydride (CaH_2) and stored over molecular sieves. High resolution mass spectrometry (HRMS) data were recorded by the mass spectrometry service of the University of Vigo, Spain.

CNB-L-Phe-OEt (4). 2-Nitrobenzyl alcohol (1.23 g, 8.00 mmol) was added portionwise over 5 minutes to a stirred solution of CDI (1.30 g, 8.00 mmol) in dry CH_2Cl_2 (20 mL). After 2 h, H-L-Phe-OEt (1.55 g, 8.00 mmol) was added and the resulting mixture was stirred for 4 h. The reaction mixture was concentrated under reduced pressure to afford a residue which was diluted with EtOAc (30 mL), and this solution was washed with $KHSO_4$ solution (1 M, 2 × 30 mL), $NaHCO_3$ solution (1 M, 2 x 30 mL) and brine (30 mL) and then dried with $MgSO_4$. Filtration followed by removal of the solvent under reduced pressure afforded a residue which was purified by flash column chromatography (30% EtOAc in hexane) to afford pure CNB-L-Phe-OEt (**4**) as a white solid (2.42 g, 81%). ^{1}H NMR ($CDCl_3$, 400 MHz) δ 1.26 (3H, t, J = 7.2, $CO_2CH_2CH_3$), 3.11 (1H, dd, J = 13.8, 6.4, β-H_AH_BPh), 3.18 (1H, dd, J = 13.8, 5.6, β-H_AH_BPh), 4.20 (2H, q, J = 7.2, $CO_2CH_2CH_3$), 4.62-4.69 (1H, m, α-C*H*CH_2Ph), 5.42 (1H, d, J = 8.0, N*H*), 5.46-5.57 (2H, m, CH_2 of CNB), 7.15 (2H, d, J = 6.8, Ar*H*), 7.24-7.30 (3H, m, Ar*H*), 7.43-7.54 (2H, m, Ar*H*), 7.61-7.65 (1H, m, Ar*H*), 8.10 (1H, d, J = 7.6, Ar*H*); ^{13}C NMR ($CDCl_3$, 100 MHz) δ 14.1 (CH_3, $CO_2CH_2CH_3$), 38.2 (CH_2, β-CH_2 of Phe), 54.8 (CH, α-CH of Phe), 61.2 (CH_2, $CO_2CH_2CH_3$), 63.4 (CH_2, CH_2 of CNB), 124.9 (CH, Ar), 127.1 (CH, Ar), 128.4 (CH, Ar), 128.5 (CH, Ar), 129.3 (CH, Ar, 131.4 (CH, Ar), 133.0 (C, Ar), 133.7 (CH, Ar), 135.7 (C, Ar), 147.2 (C, Ar), 155.0 (C, *C*=O), 171.4 (C, *C*=O); in agreement with literature data.[1]

CNB-L-Phe-OH (5). CNB-L-Phe-OEt **4** (1.70 g, 4.56 mmol) was dissolved in dioxane (46 mL) and a solution of NaOH 1 M (6.85 mL, 6.85 mmol, 1.5 equiv) was added, with stirring. The reaction was followed by TLC until no starting material was detected (typically 5 h). The organic solvent was removed under reduced pressure, and then the reaction mixture was acidified to pH 3 with $KHSO_4$ solution (1 M). The solid was filtered and washed with Et_2O. The solid was identified as CNB-L-Phe-OH **5** (1.07 g, 68%). ^{1}H NMR (DMSO-d6, 400 MHz) 2.48 (1H, dd, J = 14.0, 10.8, β-H_AH_BPh), 2.85 (1H, dd, J = 14.0, 4.4, β-H_AH_BPh), 4.11-4.20 (1H, m, α-C*H*CH_2Ph), 5.28 (2H, m, CH_2 of CNB), 7.17-7.28 (2H, m, Ar*H*), 7.47-7.59 (5H, m, Ar*H*), 7.66-7.75 (2H, m, Ar*H*, N*H*), 8.10 (1H, d, J = 6.8, Ar*H*); ^{13}C NMR (DMSO-d_6, 100 MHz) δ 36.8 (CH_2, β-CH_2 of Phe), 55.9 (CH, α-CH of Phe), 61.9 (CH_2, CH_2 of CNB), 124.7 (CH, Ar), 126.2 (CH, Ar), 128.1 (CH, Ar), 128.3 (CH, Ar), 128.8 (CH, Ar, 129.2 (CH, Ar), 133.1 (C, Ar), 134.1 (CH, Ar), 138.2 (C, Ar), 146.2 (C, Ar), 155.4 (C, *C*=O), 173.3 (C, *C*=O); in agreement with literature data.[2]

CNB-L-Phe-D,L-Phe(β-OH)-OMe (6). A stirred mixture of CNB-L-Phe-OH (**5**) (800 mg, 2.32 mmol) and H-D,L-Phe(β-OH)-OMe.HCl (538 mg, 2.32 mmol) in MeCN (20 mL) was cooled to 0 °C. HBTU (880 mg, 2.32 mmol) and triethylamine (0.96 mL, 6.92 mmol) were added sequentially, with 2 min between each addition, and then the mixture was stirred at rt overnight. Concentration under reduced pressure gave a residue that was partitioned between ethyl acetate (50 mL) and $KHSO_4$ (50 mL, 1M). After separation of the phases, the organic phase was thoroughly washed with $KHSO_4$ solution (1 M, 2 x 50 mL), $NaHCO_3$ solution (1 M, 2 × 50 mL) and brine (50 mL) and then dried with $MgSO_4$. Filtration followed by removal of the solvent under reduced pressure afforded compound **6** (1.04 g, 86 %). ^{1}H NMR (DMSO-d_6, 400 MHz) δ 2.35 and 2.65 (1H, J = 13.6, 11.2, β-CH_ACH_B Phe), 2.66 and 2.91 (^{1}H, m, J = 13.6, 4.0, β-CH_ACH_B Phe), 3.61 and 3.67 (3H, s, CO_2CH_3), 4.53-4.59 (1H, m, α-C*H* Phe), 4.62-4.83 (1H, m, β-C*H* Phe(β-OH)), 5.08-5.33 (3H, ArCH_2O, α-C*H* Phe(β-OH)), 5.97 and 6.00 (1H, d, J = 4.4, N*H*), 7.11-7.31 (8H, Ar*H*, N*H*), 7.31-7.40 (3H, m, Ar*H*), 7.51-7.59 (1H, m, Ar*H*), 7.65-7.76 (1H, m, Ar*H*), 8.05-8.10 (1H, m, Ar*H*), 8.27 and 8.42 (1H, d, J = 9.2, Ar*H*); ^{13}C NMR (DMSO-d_6, 100 MHz) δ 37.5 and 37.6 (CH_2, β-CH_2 Phe), 52.0 and 52.1 (CH_3, CO_2CH_3), 55.8 and 56.1 (CH, α-CH Phe), 58.3 and 58.5 (CH, β-CH Phe(β-OH), 62.0 and 62.2 (CH_2, ArCH_2O) 72.2 and 72.3 (CH, α-*CH* Phe(β-OH), 124.8 (CH, Ar), 126.18 and 126.22 (CH, Ar), 126.3 and 126.4 (CH, Ar), 127.2 and 127.4 (CH, Ar), 127.7 and 127.8 (CH, Ar), 127.9 and 128.0 (CH, Ar), 128.1 and 128.2 (CH, Ar), 128.7 and 128.8 (CH, Ar), 129.3 (CH, Ar), 133.1 and 133.2 (C, Ar), 134.13 and 134.15 (CH, Ar), 137.9 and 138.1 (C, Ar), 141.57 and 141.61 (C, Ar), 146.78 and 146.83 (C, Ar), 155.19 and 155.21 (C, *C*=O) 170.7 (C, *C*=O), 171.79 and 171.83 (C, *C*=O); HRMS *m/z* (EI) 522.1869 ($[M+H]^+$). $C_{27}H_{28}ON_3O_8$ requires 522.1876.

CNB-L-Phe-Z-ΔPhe-OMe (7). DMAP (23 mg, 0.19 mmol) and Boc_2O (417 mg, 1.91 mmol) were added to a solution of compound **6** (1.00 g, 1.91 mmol) in dry MeCN (5 mL) under rapid stirring at rt. The mixture was monitored by ^{1}H NMR and stirred at rt until all reactant was consumed (typically 5 h). *N,N,N′,N′*-tetramethylguanidine (2.0 % in volume, 0.10 mL) was added under continued stirring. The mixture was monitored by ^{1}H NMR and stirred at rt until all of the intermediate was consumed (typically 3 h). Concentration under reduced pressure gave a residue that was partitioned between ethyl acetate (30 mL) and $KHSO_4$ solution (1 M, 30 mL). After separation of the phases, the organic phase was washed with $KHSO_4$ solution (1 M, 2 x 30 mL), $NaHCO_3$ solution (1 M, 2 × 30 mL), water (1 × 30 mL) and brine (30 mL) and then dried with $MgSO_4$. Filtration followed by removal of the solvent under reduced pressure afforded compound **7** (781 mg, 81%). ^{1}H NMR (DMSO-d6, 400 MHz) δ 2.81 (1H, dd, J = 13.6, 11.2, β-C$H_A$$H_B$ Phe), 3.15 (1H, dd, J = 13.6, 3.6, β-C$H_A$$H_B$ Phe), 4.38-4.44 (1H, m, α-C*H* Phe), 5.33 (2H, s, ArCH_2O), 7.20-7.42 (9H, m, Ar*H*, β-C*H* ΔPhe,), 7.50 (1H, d, J = 8.0, Ar*H*), 7.56 (1H, t, J = 7.6, Ar*H*), 7.64 (2H, dd, J = 6.0, 3.6, Ar*H*), 7.70 (1H, td, J = 7.6, 1.2, Ar*H*), 8.02 (1H, br s, N*H*), 8.10 (1H, dd, J = 8.0, 0.8, Ar*H*), 1

x N*H* was not observed; ^{13}C NMR (DMSO-d_6, 100 MHz) δ 36.9 (CH_2, β-CH_2 of Phe), 52.2 (CH, α-*C*H of Phe), 56.3 (CH_3, CO_2CH_3), 62.0 (CH_2, *CH_2* of CNB), 124.7 (CH, Ar), 126.4 (CH, Ar), 128.1 (CH, Ar), 128.3 (CH, Ar), 128.5 (CH, Ar), 128.6 (C, -*C* Phe), 128.8 (CH, Ar), 129.3 (CH, Ar), 129.5 (CH, Ar), 129.8 (CH, Ar), 130.2 (CH, Ar), 132.1 (CH, Ar), 133.0 (C, Ar), 133.2 (C, Ar), 134.1 (CH, Ar), 138.0 (C, Ar), 146.9 (C, Ar), 155.6 (C, *C*=O), 165.3 (C, *C*=O), 171.4 (C, *C*=O); HRMS m/z (EI) 504.1765 ($[M+H]^+$). $C_{27}H_{26}N_3O_7$ requires 504.1771.

CNB-L-Phe-Z-ΔPhe-OH (2). The dehydrodipeptide **7** (500 mg, 0.98 mmol) was dissolved in dioxane (9.8 mL) and a solution of NaOH 1 M (1.47 mL, 1.47 mmol, 1.5 equiv) was added. The reaction was followed by TLC until no starting material was detected (typically 5 h). The organic solvent was removed under reduced pressure, and the reaction mixture was acidified to pH 3 with $KHSO_4$ solution (1 M). The solid was filtered and washed with Et_2O. The solid was identified as CNB-L-Phe-Z-ΔPhe-OMe (**2**) (279 mg, 58%). ^{1}H NMR (DMSO-d_6, 400 MHz) δ 2.81 (1H, dd, J = 13.6, 11.2, β-H_AH_BPh), 3.15 (1H, dd, J = 13.6, 3.6, β-H_A*H_B*Ph), 4.40-4.47 (1H, m, α-C*H*CH_2Ph), 5.26-5.35 (2H, m, C*H_2* of CNB), 7.17-7.42 (9H, m, Ar*H*, β-C*H* ΔPhe, N*H* Phe), 7.46 (1H, d, J = 7.6, ArH), 7.47-7.58 (3H, m, Ar*H*), 7.68 (1H, dd, J = 9.2, 8.8, Ar*H*), 7.87 (1H, d, J = 9.0, Ar*H*), 8.09 (1H, dd, J = 9.0, 0.8, Ar*H*), 9.67(1H, s, N*H* ΔPhe); ^{13}C NMR (DMSO-d_6, 100 MHz) δ 37.0 (CH_2, β-*CH_2* of Phe), 56.6 (CH, α-CH of Phe), 62.1 (CH_2, *CH_2* of CNB), 124.8 (CH, Ar), 126.4 (CH, Ar), 127.4 (C, α-*C* ΔPhe), 128.2 (CH, Ar), 128.3 (CH, Ar), 128.4 (CH, Ar), 128.8 (CH, Ar), 128.9 (CH, Ar), 129.4 (CH, Ar), 129.9 (CH, Ar), 133.1 (C, Ar), 134.1 (C, Ar), 134.2 (CH, β-CH ΔPhe), 138.2 (C, Ar), 146.9 (C, Ar), 155.7 (C, *C*=O), 166.8 (C, *C*=O), 171.0 (C, *C*=O); HRMS *m/z* (EI) 490.1609 ($[M+H]^+$). $C_{26}H_{24}N_3O_7$ requires 490.1614.

Boc-L-Phe-D,L-Phe(β-OH)-OMe (9). A mixture of Boc-L-Phe-OH (**8**) (1.29 g, 4.88 mmol) and H-D,L-Phe(β-OH)-OMe.HCl (1.13 g, 4.88 mmol) in MeCN (15 mL) and cooled to 0 °C. HBTU (2.78 g, 5.86 mmol) and triethylamine (1.51 mL, 11.0 mmol) were added sequentially, with 2 min between each addition, and then the mixture was stirred at rt overnight. Concentration under reduced pressure gave a residue that was partitioned between ethyl acetate (30 mL) and $KHSO_4$ solution (30 mL, 1 M). After separation of the phases, the organic phase was thoroughly washed with $KHSO_4$ solution (1 M, 2 × 30 mL), $NaHCO_3$ solution (1 M, 2 × 30 mL) and brine (30 mL) and then dried with $MgSO_4$. Filtration followed by removal of the solvent under reduced pressure afforded compound **9** (2.15 g, 92%). ^{1}H NMR ($CDCl_3$, 400 MHz) δ 1.39 (9H, s, CO_2C(C*H_3*)$_3$), 2.79-2.94 (1H, m, β-C*H_A*H_BPh Phe), 2.95-3.06 (1H, m, β-CH_A*H_B*Ph Phe), 3.87 and 3.71 (3H, s, CO_2C*H_3*), 3.42 and 3.52 (1H, brs and d, J = 4.4, O*H*), 4.21-4.44 (1H, m, *α*-C*H* Phe), 4.72-4.78 (1H, m, *α*-C*H* Phe(β-OH), 4.91-5.04 (1H, m, N*H*), 5.18-5.29 (1H, m, β-C*H* Phe(β-OH)), 6.80-6.89 (1H, m, N*H*), 7.18.7.35 (10H, m, Ph*H*). ^{13}C NMR ($CDCl_3$, 100 MHz) δ 28.2 (3 x CH_3, OC(CH_3)$_3$), 38.1 (CH_2, β-CH_2 Phe), 52.5 and 52.6 (CH_3, CO_2CH_3), 55.1 and 55.5 (CH, α-CH Phe), 58.2 and 58.3 (CH, β-CH Phe(β-OH)), 73.3 and 73.7 (CH, α-CH Phe(β-OH), 80.1 (C, O*C*(CH_3)$_3$), 125.8 and 125.9 (CH, Ph), 126.9 and 126.7 (CH, Ph), 127.97 and 128.04 (CH, Ph), 128.3 (CH, Ph), 128.4 and 128.5 (CH, Ph), 129.2 (CH, Ph), 136.3 and 136.5 (C, Ph), 139.5 and 139.6 (C, Ph), 155.5 (C, *C*=O), 170.5 and 170.8 (C, *C*=O), 171.6 and 171.7 (C, *C*=O); in agreement with literature data.[3]

Boc-L-Phe-Z-ΔPhe-OMe (10). DMAP (54 mg, 0.44 mmol) and Boc_2O (961 mg, 4.35 mmol) were added to a solution of compound **9** (2.15 g, 4.35 mmol) in dry MeCN (12 mL, 1 M), under rapid stirring at rt. The reaction was monitored by ^{1}H NMR and stirred at rt until the reactant was consumed (typically 5 h). *N,N,N′,N′*-tetramethylguadinine (2 % in volume, 0.24 mL) was added under continued stirring. The mixture was monitored by ^{1}H NMR and stirred at rt until all of the intermediate was consumed (typically 3 h). Concentration under reduced pressure gave a residue that was partitioned between ethyl acetate (50 mL) and $KHSO_4$ (1 M, 30 mL). After separation of the phases, the organic phase was washed with $KHSO_4$ (1 M, 2 × 30 mL), $NaHCO_3$ (1 M, 2 x 30 mL), water (30 mL) and brine (30 mL) and then dried with $MgSO_4$. Filtration followed by removal of the solvent under reduced pressure afforded compound **10** (1.69 g, 77%). ^{1}H NMR ($CDCl_3$, 400 MHz,) δ 1.41 (9H, s, CO_2C(C*H_3*)$_3$), 3.08 (1H, dd, J = 14.0 Hz, 7.2 Hz, β-C*H_A*CH_BPh Phe), 3.21 (1H, dd, J = 14.0 Hz, 6.4 Hz, β-CH_AC*H_B*Ph Phe), 3.82 (3H, s, CO_2C*H_3*), 4.46-4.55 (1H, m, *α*-C*H* Phe), 4.97 (1H, br s, N*H*), 7.28-7.42 (11H, m, Ph*H*, *α*-C*H*Phe), 7.68 (1H, br s, NH); ^{13}C NMR ($CDCl_3$, 100.6 MHz) δ: 28.1 (3 x CH_3, OC(CH_3)$_3$), 37.5 (CH_2, β-CH_2 Phe), 52.5 (CH_3, CO_2CH_3), 80.3 (C, O*C*(CH_3)$_3$), 123.8 (C, α-*C* ΔPhe), 126.8 (CH, Ph), 128.49 (CH, Ph), 128.53 (CH, Ph), 129.3 (CH, Ph), 129.4 (CH, Ph), 129.7 (CH, Ph), 132.7 (CH, β-CH ΔPhe), 133.3 (C, Ph), 136.4 (C, Ph), 165.3 (C, *C*=O ΔPhe), 170.3 (C, *C*=O Boc), 171.1 (C, *C*=O Phe); in agreement with literature data.[3]

H-L-Phe-Z-ΔPhe-OH (3). *Method 1* (from Boc-L-Phe-Z- Phe-OMe (**10**)). Compound **10** (423 mg, 0.99 mmol) was dissolved in dioxane (9.90 mL) and a solution of NaOH 1 M (1.49 mL, 1.49 mmol, 1.5 equiv) was added. The reaction was followed by TLC until no starting material was detected (typically 5 h). The organic solvent was removed under reduced pressure, and the reaction mixture was acidified to pH 3 with $KHSO_4$ (1 M). The solid was filtered and washed with Et_2O. After removal of the residual Et_2O, the solid was dissolved in TFA (3.0 mL) and the reaction mixture was stirred at room temperature for 30 min. The TFA was then removed under reduced pressure. Traces of residue TFA were removed by the addition and removal of $CHCl_3$ (3 x 10 mL) under reduced pressure, affording H-L-Phe-Z-ΔPhe-OH (**3**) as a white solid (237 mg, 54%).

Method 2 (from CNB-L-Phe-Z-ΔPhe-OH (2)). A solution of compound **2** (100 mg, 0.204 mmol) in MeCN (5 mL) was irradiated with UV light (360 nm) for 3 h. After cooling to 0 °C, the precipitate formed was filtered and the solid was then washed with cooled (0 °C) MeCN (2 × 5 mL). Removal of the residual MeCN under reduced pressure afforded H-L-Phe-*Z*-ΔPhe-OH (**3**) as a white solid (48 mg, 76%). The ^{1}H NMR and ^{13}C NMR data were identical to that from the material obtained from method 1.

^{1}H NMR (DMSO-d_6, 400 MHz) δ 2.92 (1H, dd, J = 13.9, 7.0, β-C*H_A*CH_BPh Phe), 3.06 (1H, dd, J = 13.9, 5.2, β-CH_AC*H_B*Ph Phe), 4.35-4.41 (1H, m, α-C*H* Phe), 6.58 (1H, d, J = 7.6, NI_2), 6.94 (1H, s, β-C*H* ΔPhe), 7.12-7.7.41 (8H, m, Ph*H*),

7.52 (2H, d, J = 6.8, Ph*H*), 7.98 (1H, d, s, N*H* ΔPhe); ^{13}C NMR (DMSO-d_6, 100 MHz) δ 37.4 (CH_2, β-CH_2 Phe), 53.8 (CH, α-*C*H Phe), 126.5 (CH, Ph), 126.7 (CH, β-*C*H ΔPhe), 128.2 (CH, Ph), 128.3 (CH, Ph), 128.4 (CH, Ph), 129.4 (CH, Ph), 129.5 (CH, Ph), 134.3 (C, Ph), 137.2 (C, Ph), 154.7 (C, α-*C* ΔPhe), 167.1 (C, *C*=O), 173.4 (C, *C*=O); HRMS *m*/*z* (EI) 311.1388. $C_{18}H_{19}N_2O_3$ requires 311.1396.

References for Synthetic Chemistry Section:

1. Y. Ishizuka, J. Chem. Soc. Japan, **1956**, 77, 90–93.
2. A. Stutz, C. Höbartner and S. Pitsch, Helv. Chim. Acta, **2000**, 83, 2477–2503.
3. P. M. T. Ferreira, L. S. Monteiro and G. Pereira, Amino Acids, **2010**, 39, 499–513.

UV-promoted hydrogel dissolution study in contact with simulated biological fluid (SBF)

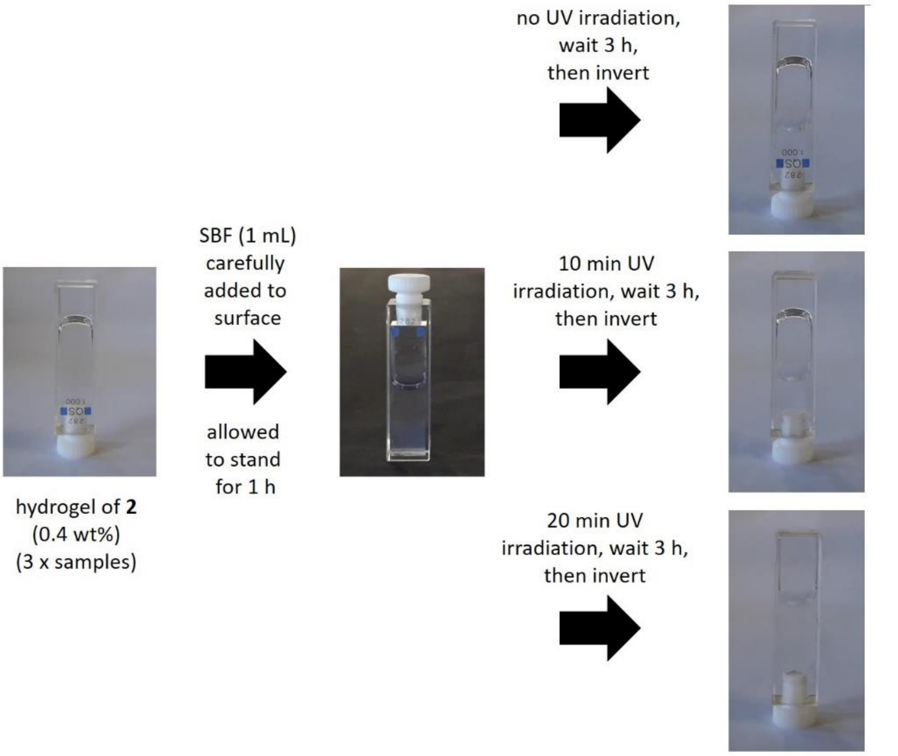

Figure S3: Visual study of gel-to-sol transition of CNB-Phe-ΔPhe-OH hydrogel in contact with SBF.

Reference for SBF preparation: T. Kokubo and H. Takadama, Biomaterials, **2006**, 27, 2907–2915.

<u>HPLC study of the UV-promoted conversion of compound **2** to compound **3** in the gel state</u>

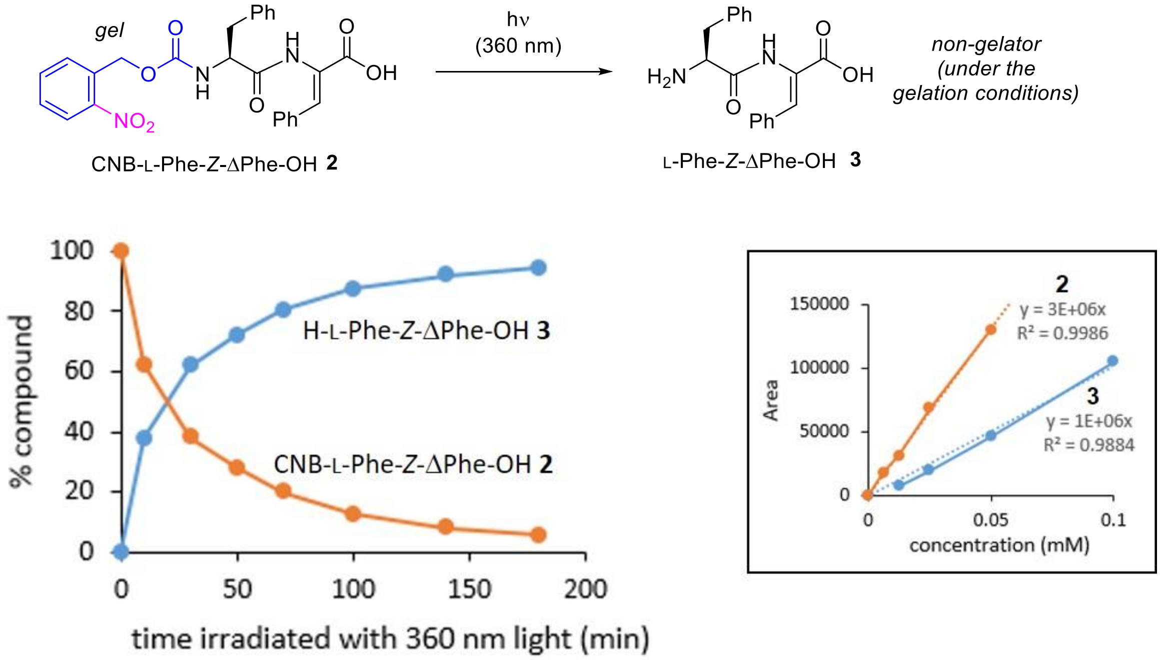

Figure S4: Top: Photo-catalysed formation of **3** from **2**. Left: The UV-promoted conversion of compound **2** into compound **3** followed by HPLC. Right: Calibration graph (for determining relative response factors) for compounds **2** and **3**.

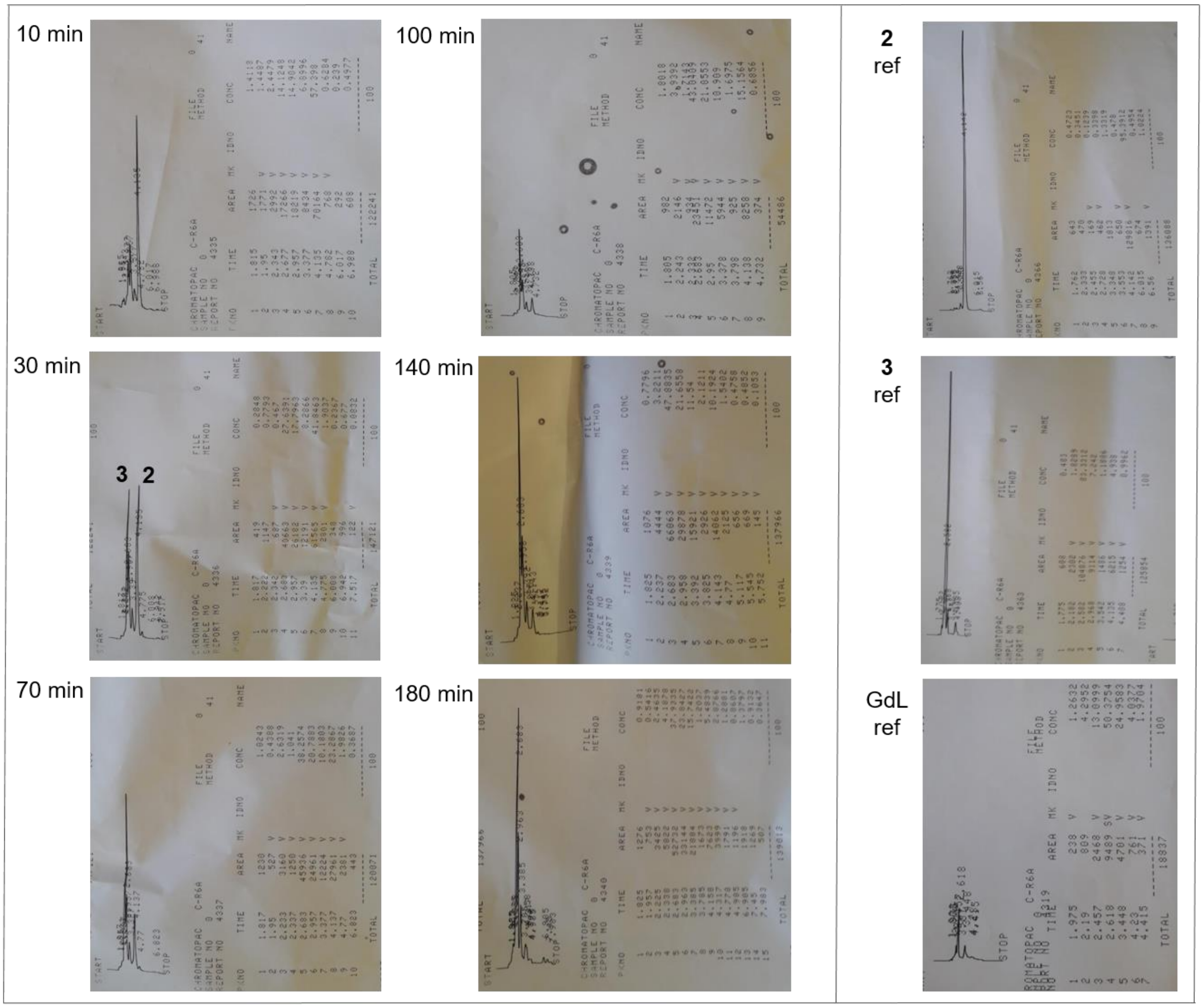

Figure S5: HPLC traces showing the conversion of compound **2** to compound **3**. Traces for reference samples of **2**, **3** and GdL are also shown.

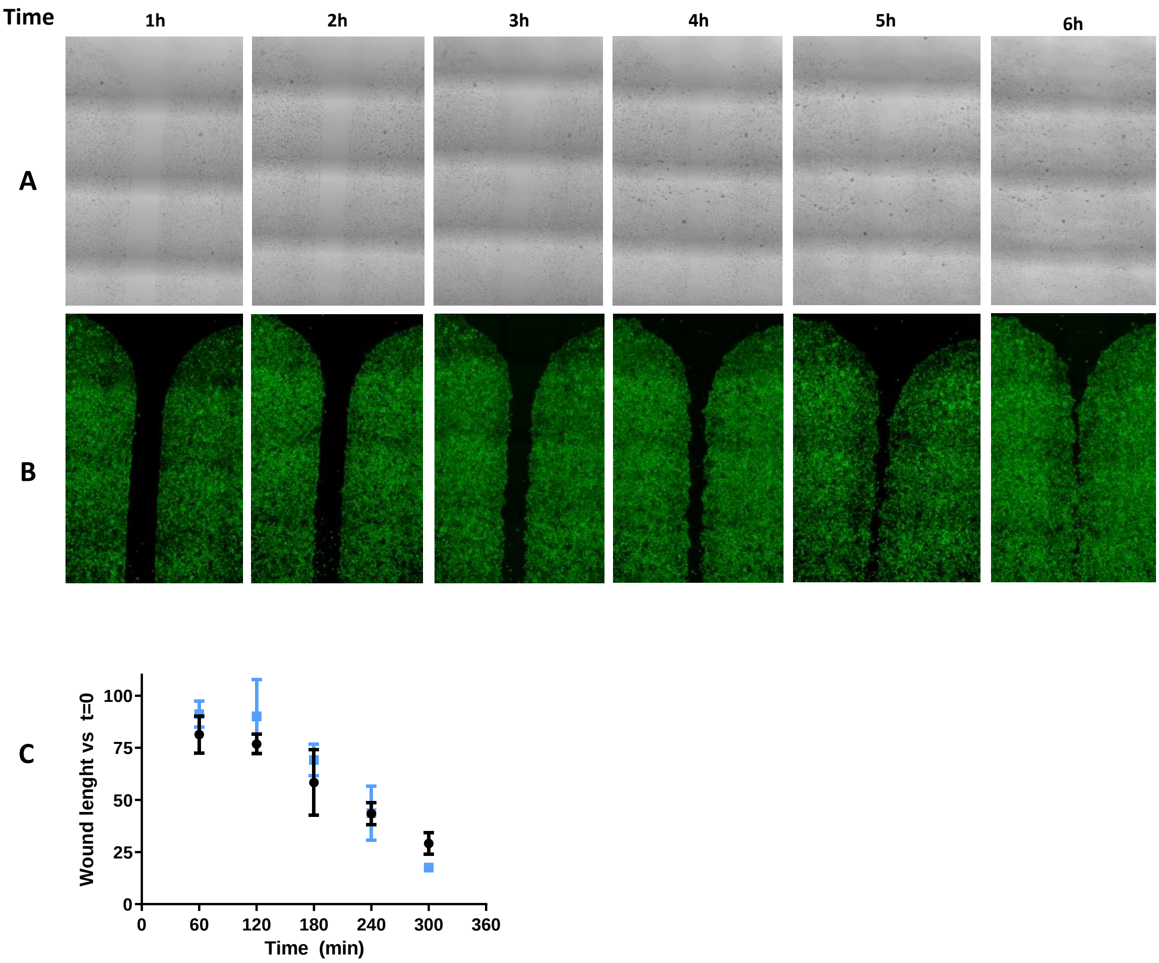

Figure S6. (**A**) Brightfield mosaic of several fields obtained with the 10x objective. (**B**) Processed images, resulting from the transformation of raw images with the software's built-in "edge" macro and green LUT. (**C**) Width of wound gap though time, when compared with t = 0 (maximum width). Black series: control; blue series: cells incubated with H-L-Phe-Phe-OH **3** at 100 μM.

In Vitro Drug Delivery Studies

Drug delivery simulation studies were carried on gels of CNB-Phe-ΔPhe-OH **2**, to determine if a short treatment with UV light could stimulate an increased rate of release of model drug compound from within a disrupted, or partially disrupted, gel matrix. Following an adaptation of a method described by Abraham *et al.*,[10.1039/D0TB01157F] the dyes methylene blue and methyl orange were used as examples of negatively and positively charged model drug compounds, respectively, whose release could be studied by measuring the absorbance at their respective $_{max}$ values of 666 nm and 486 nm. The antimicrobial compound, ciprofloxacin, was used as an example of an overall neutral compound, whose release was studied by HPLC.[10.1039/C5TB01820J]

Gels of CNB-Phe-ΔPhe-OH **2** (1.0 mL, 0.4 wt%) were prepared in a UV cuvette as described in Section 2.2 (except that the water was switched to 0.20 mmol methylene blue solution, 0.10 mmol methyl orange solution or 0.20 mmol ciprofloxacin solution) and allowed to stand overnight, then 1.5 mL of water was carefully layered on the surface. The cumulative percentage of model drug compound released (see Materials and Methods section for full details), with and without exposure to UV light, was measured versus time. We investigated the effect of both 10 minute- and 20 minute-exposures to UV (360 nm).

In the absence of UV light, the percentage release of methylene blue reached a plateau of ~4.5% after 48 hours, which stayed constant until 96 hours. The low percentage release is expected for the release of a positively charged compound from a negatively charged hydrogel[10.1039/D0TB01157F]. Applying a 10-minute dose of UV irradiation (360 nm) slightly increased the amount of methylene blue release, with a new plateau at 5.2% being reached over the next 24 hours (192 hours total). In a separate experiment, applying a 10 min dose of UV light (360 nm) at three hours resulted in a slightly increased overall percentage release relative to the control, with a plateau of just over 5% being reached (Figure 11, A and B). In a third experiment, applying a 20 min dose of UV light (360 nm) at 3 hours provided a complete release of methylene blue at 24 hours owing to complete gel dissolution.

Repeating the experiment with gels containing methyl orange gave higher levels of compound release. A plateau of 54% release was recorded after 96 hours, which was maintained until 168 hours. A 10-minute treatment with UV light (360 nm) then produced an extra release of methyl orange, and a new plateau of 67% release was reached 48 hours later (216 hours total). In a separate experiment, UV irradiation at 3 hours produced a similar maximum transfer level, reaching 64% release after 96 hours, versus 54% for the control (Figure 11, C and D). A separate experiment where the gel was exposed to UV light (360 nm) for 20 minutes provided a complete dissolution of the hydrogel, and therefore a quantitative release of methyl orange.

Finally, ciprofloxacin was investigated as an example of an antimicrobial compound, in an experiment monitored by HPLC. In the absence of UV light, no ciprofloxacin was released within the limits of HPLC detection, even after 168 hours. The equivalent experiment was then performed where a 10-minute treatment with UV light (360 nm) was given at 3 hours. In this case, ciprofloxacin release was stimulated, reaching ~6% release after 168 hours (Figure 11, E and F). Finally, an experiment where a 20-minute exposure to UV light (360 nm) was made after 3 hours provided a quantitative release of ciprofloxacin after 24 hours.

The fact that a UV irradiation time of 20 minutes resulted in the eventual complete dissolution of the gel structure in each case (accompanied by complete drug release), whereas the gels appeared completely intact following an shorter UV irradiation time of 10 minutes (a small increase in drug release relative to control), suggests that within this window there is the potential for an intermediate UV exposure to allow tuned drug release. When we consider the HPLC study of the photo-cleavage of CNB-Phe-ΔPhe-OH (**2**) presented in Section 3.4, and the gelation studies in Section 3.2, it is no surprise that a 20 minute UV exposure would take the concentration of gelator below the CGC.

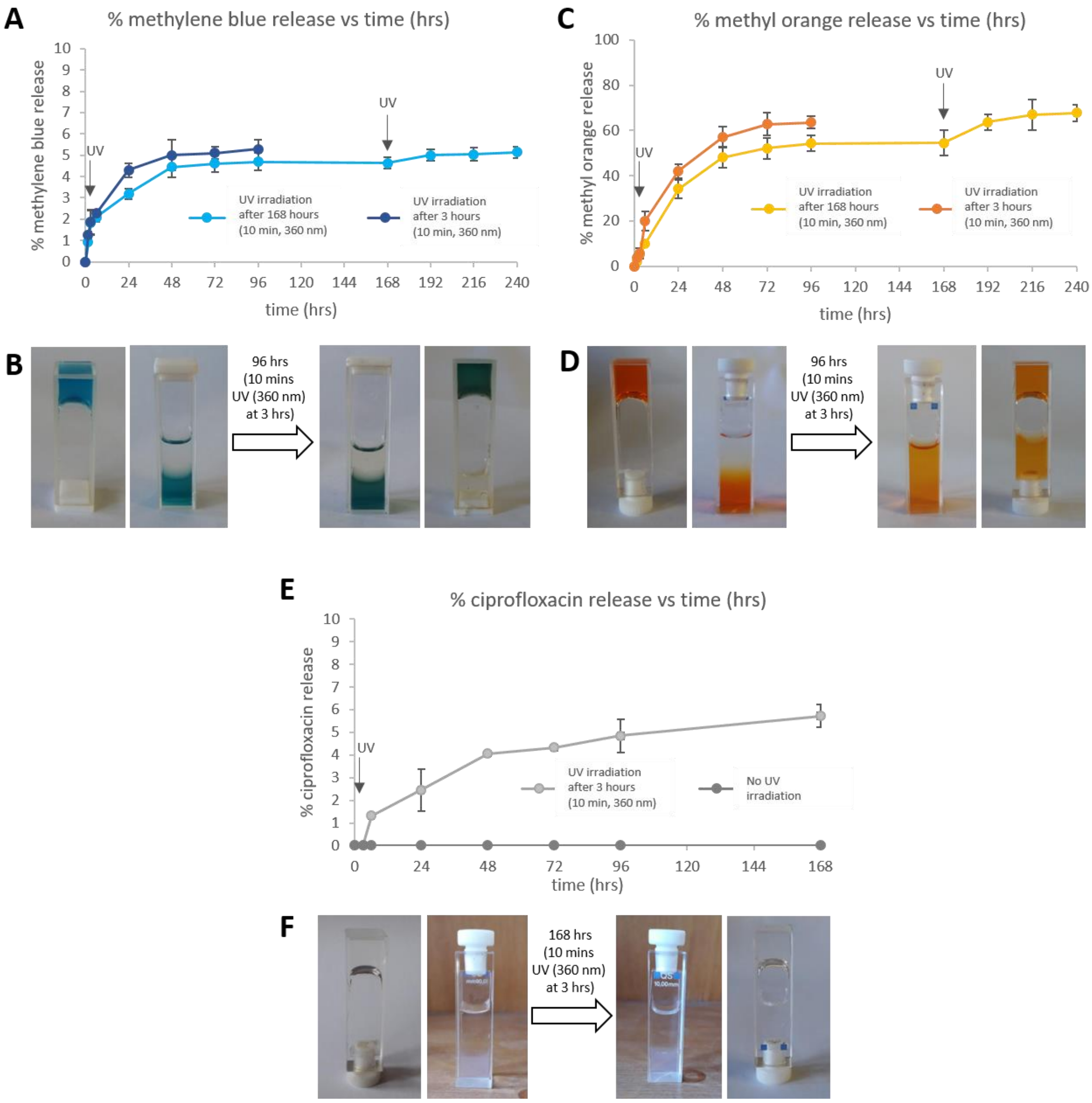

Figure S7. (**A**) Graph showing percentage release of methylene blue from hydrogel of 2 when irradiated with UV light (360 nm) for 10 minutes after 168 hours (light blue line) or after 3 hours (dark blue line). (**B**) Photographs showing the appearance of the methylene blue hydrogel experiment at the start and the end of the experiment. (**C**) Graph showing percentage release of methyl orange from hydrogel of 2 when irradiated with UV light (360 nm) for 10 minutes after 168 hours (light orange line) or after 3 hours (dark orange line). (**D**) Photographs showing the appearance of the methyl orange hydrogel experiment at the start and the end of the experiment. (**E**) Graph showing percentage release of ciprofloxacin from a hydrogel of 2 when irradiated with UV (360 nm) for 10 minutes after 3 hours (light grey line) or without any UV irradiation (dark grey line). (**F**) Photographs showing the appearance of the ciprofloxacin hydrogel experiment at the start and the end of the experiment.
